# Supplementary material for: Cryo-electron microscopy of the f1 filamentous phage reveals insights into viral infection and assembly
Source: Nat Commun. 2023 May 11;14:2724. doi: 10.1038/s41467-023-37915-w (PMC10175506; doi:10.1038/s41467-023-37915-w)
Supplement: Supplementary file 1 — Supplementary Information [file 41467_2023_37915_MOESM1_ESM.pdf]

Cryo-electron microscopy of the f1 filamentous phage reveals insights into viral infection and assembly

Rebecca Conners, Rayén Ignacia León-Quezada, Mathew McLaren, Nicholas J Bennett, Bertram Daum, Jasna Rakonjac & Vicki A M Gold

Supplementary Information

Supplementary Figures

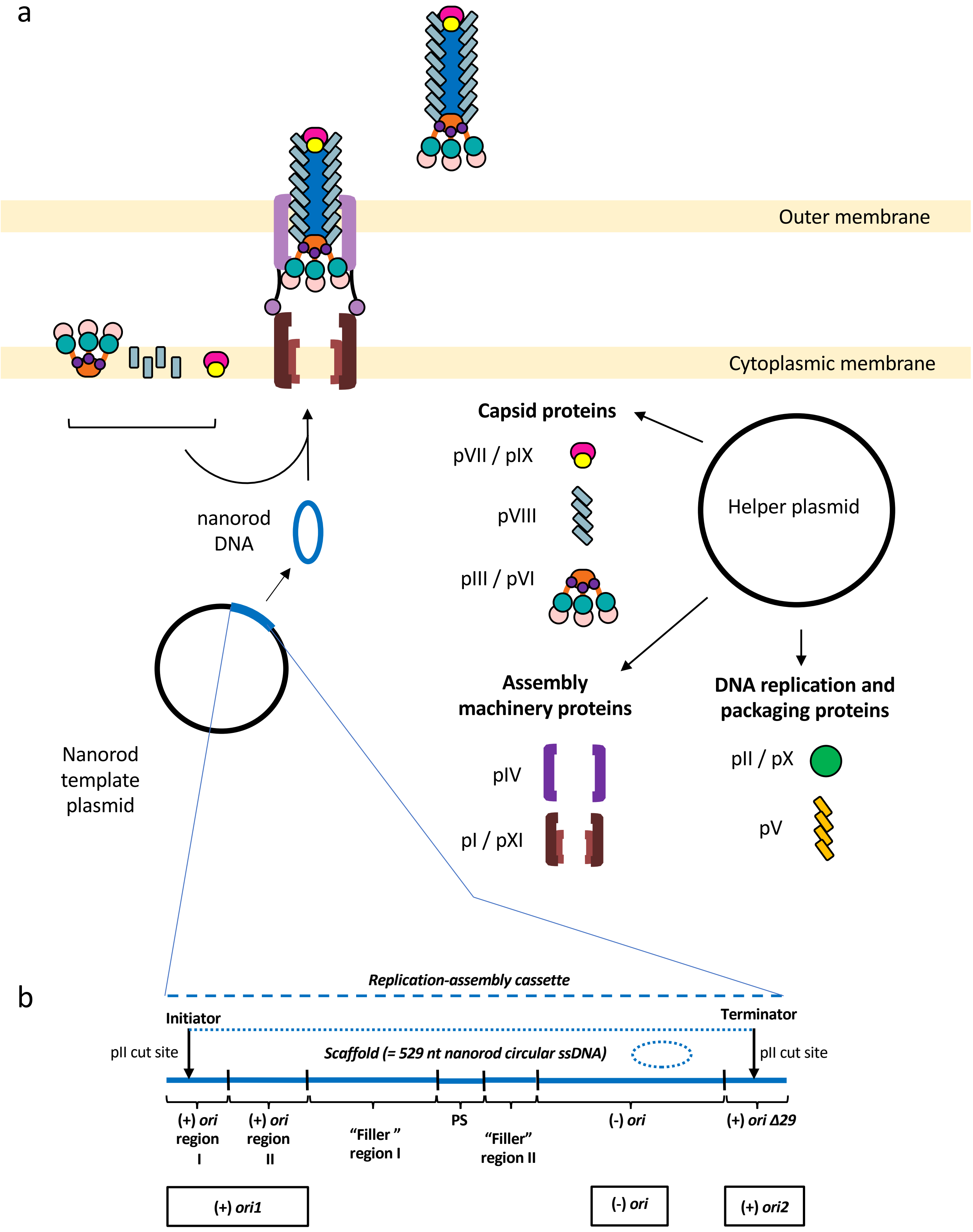

Supplementary Figure 1. Schematic of the nanorod production system.

(a) Schematic drawing of the two-plasmid-based nanorod production system. The system is comprised of a nanorod template plasmid (pBSFpn529) and a helper plasmid (pHP1YM). pBSFpn529 contains a replication-assembly

cassette, that in the presence of pHP1YM, is replicated to form circular ssDNA of 529 nucleotides, the backbone of the nanorod. The helper plasmid encodes all phage proteins required for replication and assembly of the nanorods. The capsid proteins are coloured as per Fig. 1. The pI/pXI complex in the cytoplasmic membrane is shown in alignment with the outer membrane secretin pIV to allow phage egress.

(b) Nanorod replication-assembly cassette within the pBSFpn529 plasmid. “Scaffold” indicates the sequence that is replicated to generate the (+) strand circular ssDNA forming the backbone of the nanorods, between the Ff replicator protein pII cut sites within the replication-assembly cassette. (+) *ori1* corresponds to the complete positive origin of replication, encompassing regions I and II; (-) *ori*, negative strand origin of replication, (+) *ori2*, truncated (+) origin of replication (*Δ29*) encompassing truncated *ori* region I (containing a deletion of 3’-terminal 29 nt) that serves as the terminator of ssDNA replication. “Filler” indicates DNA segments between the functional sequences. The design of this cassette that includes the (-) *ori* allows replication of both strands once the ssDNA has been excised. This in turn results in an increased nanorod production in comparison to the previously reported nanorod template plasmids containing only the (+) *ori* that allows only the positive strand replication from the template plasmid[1-3].

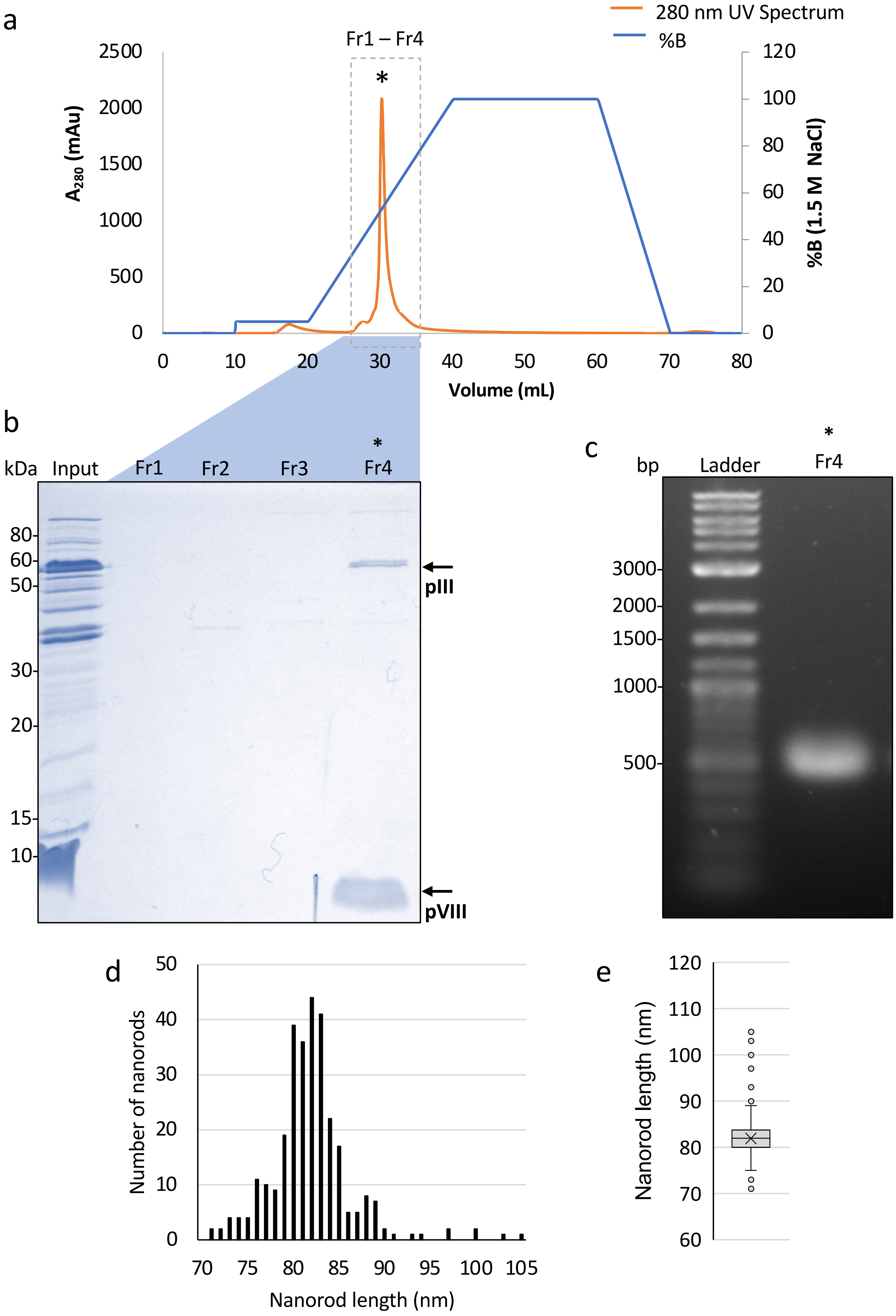

Supplementary Figure 2. Purification of nanorods by ion-exchange chromatography.

Nanorod samples partially purified by CsCl density gradient centrifugation were subjected to chromatography using a SepFast™ Supor Q column (Strong anion Q, -N+$(CH_3)_3$). The experiment was conducted at least 3 times with similar results.

(a) Chromatogram depicting UV absorption (mAu; milli Absorption units) at 280 nm (orange line) and %B, corresponding to the salt concentration gradient (blue line, from 0 to 1.5 M NaCl).

(b) 16% Tris-glycine SDS-PAGE of the collected fractions. Lanes: kDa, sizes of the molecular weight standard from Novex Sharp pre-stained marker; Input, sample before chromatography (CsCl-purified nanorods); Fr1, Fr2, Fr3 and Fr4 are fractions corresponding to the peak indicated in (a). Nanorod proteins pIII and pVIII are indicated.

(c) Fr4 was dialysed and concentrated. To confirm the presence of nanorods, they were disassembled by heating at 100 °C for 10 min, and the released DNA was analysed by agarose gel (1.2%) electrophoresis and visualised by staining with ethidium bromide. Lanes: Ladder, 1 Kb plus ladder (a double-stranded liner DNA standard used as a signpost for migration due to the lack of appropriate circular ssDNA standards; numbers indicate sizes of the standard bands in base-pairs, bp); Fr4, the fraction from the ion exchange chromatography (see b). The final nanorod concentration was 2.2 ± 0.2 mg/ml.

(d) Histogram of the nanorod length distribution plotted from the length measurements of 300 well-separated particles imaged by negative stain electron microscopy as described in Supplementary Methods. The sample size was based on statistical considerations adopted for certification of nanomaterials (European Commission, Joint Research Centre Directorate F – Health, Consumers and Reference Materials, Geel, Belgium) used to assess dispersity of particles in the nanorod standards by TEM[4].

(e) Box and whiskers plot showing distribution of the 300 nanorod lengths shown in (d). 50% of nanorods (box) are between 80 and 84 nm, and 97% between 75 and 89 nm long. The box values correspond to: top line, upper quartile; mid-line, median; bottom line, lower quartile. Whiskers correspond to maximum and minimum, apart from outliers. The range of outliers above the maximum is wider than the one below the minimum, likely due to the lateral pairing (interaction or sticking) of the nanorods, with a pair of nanorods giving an appearance of one longer nanorod.

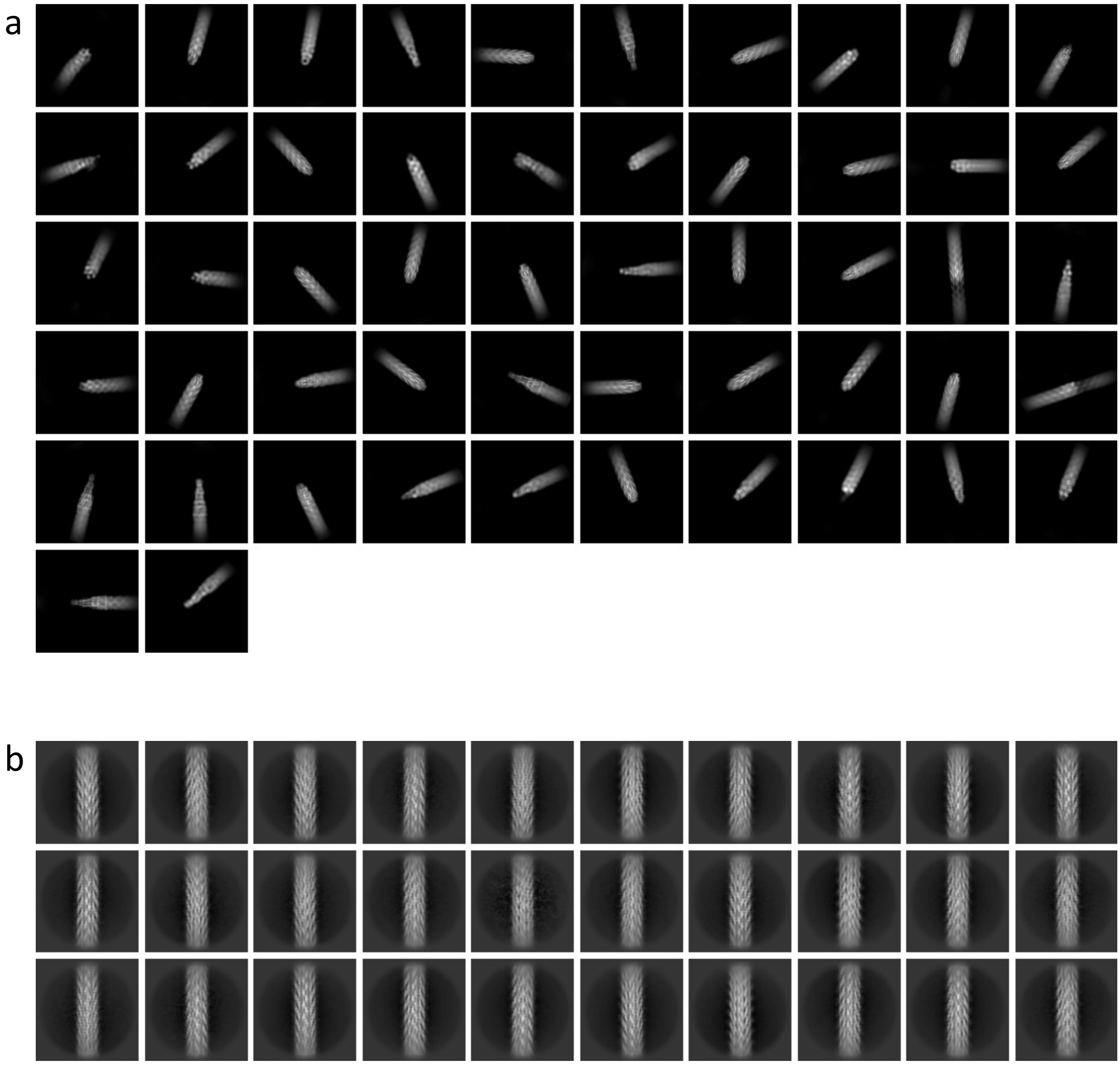

Supplementary Figure 3. 2D class averages.

(a) 2D class averages from cryoSPARC for the round and pointy tips.

(b) 2D class averages from cryoSPARC for the central, filamentous section.

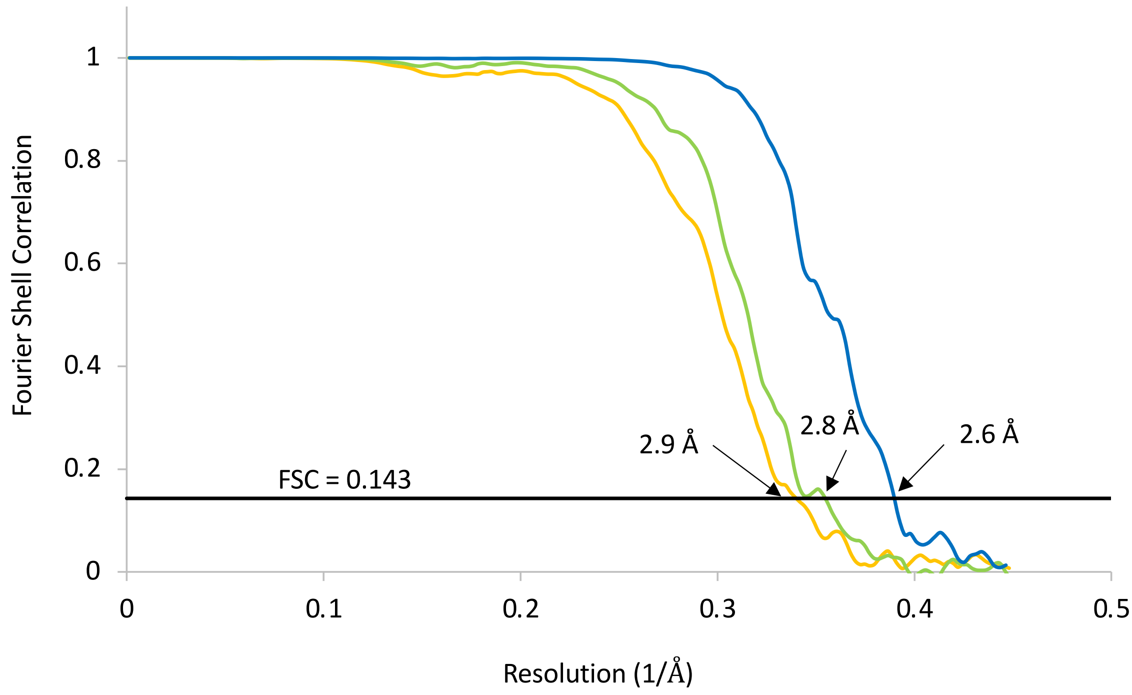

Supplementary Figure 4. FSC curves.

Gold-standard Fourier Shell Correlation (FSC) curves indicate overall resolutions of 2.9 Å, 2.8 Å and 2.6 Å for the pointy tip (yellow), round tip (green) and central filamentous part of the nanorod (blue) respectively, at FSC = 0.143.

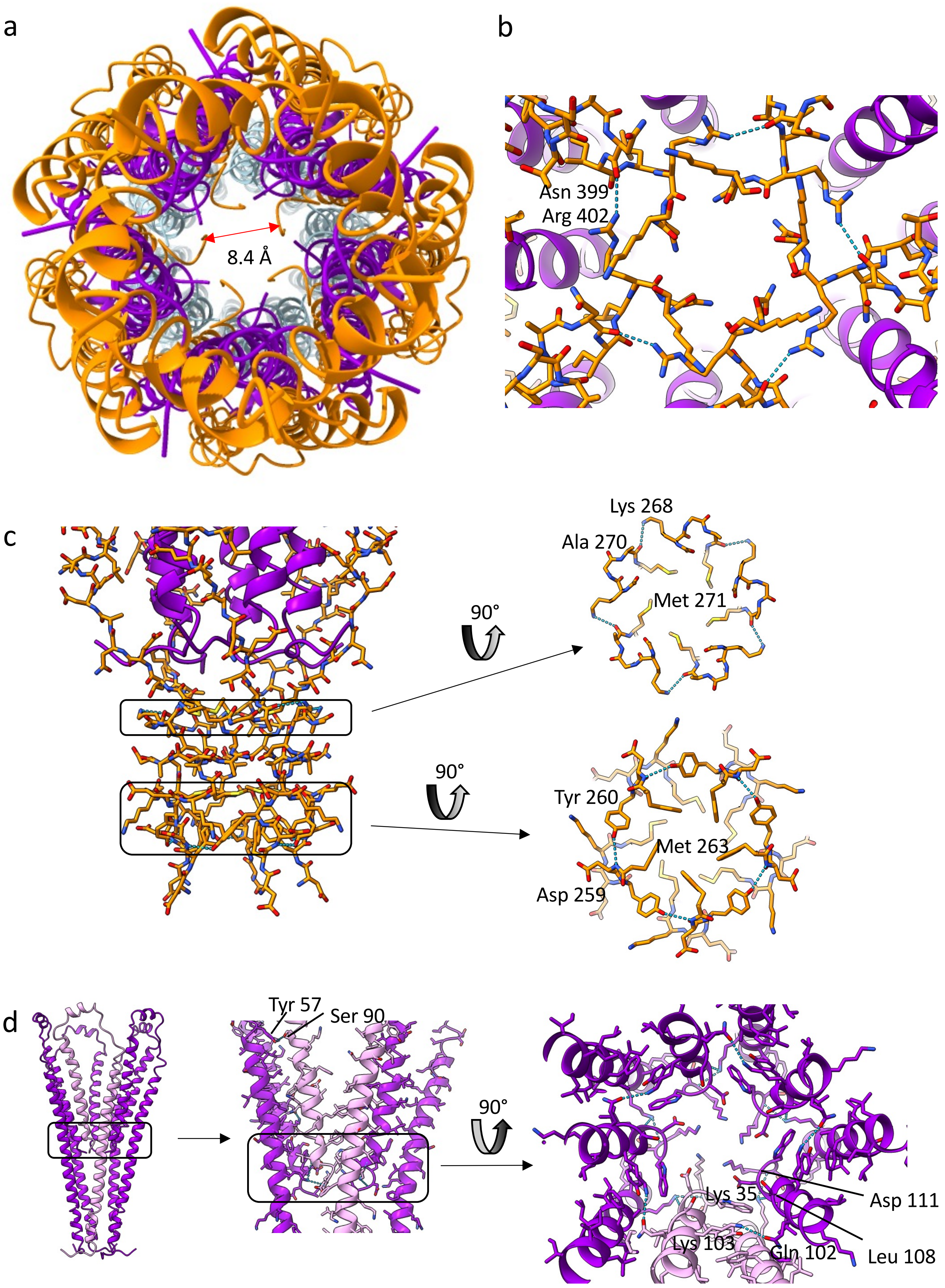

Supplementary Figure 5. Molecular interactions in pIII and pVI at the pointy tip.

(a) The C-termini of pIII proteins (orange) are buried in the centre of the phage, with the 5 symmetrical copies forming a stricture of 8.4 Å diameter. pVI is shown in purple. The diameter of the lumen at its widest point in the central region is 25 Å.

(b) At the C-terminus of pIII, a hydrogen bond is formed between the sidechain of Arg 402 and the mainchain carbonyl oxygen of Asn 399 from a neighbouring pIII chain. pIII is shown as sticks, pVI as ribbons, and the 5 hydrogen bonds in the pentamer as blue dashed lines.

(c) pIII interchain hydrogen bonds in the pointy tip. Hydrogen bonds are formed between the carbonyl oxygen of Ala 270 and the sidechain of Lys 268 from the neighbouring pIII chain. This hydrogen bonding occurs near a ring of 5 methionine sidechains (Met 271; 1 from each from each pIII). Moving nearer the tip, hydrogen bonds are formed between the sidechain of Tyr 260 and the backbone amide of Asp 259, again near a ring of 5 methionine sidechains (Met 263; 1 from each pIII).

(d) pVI pentamer shown in purple, with one chain in lilac. pVI interchain hydrogen bonds are formed between Tyr 57 and Ser 90 of a neighbouring pVI molecule. All remaining interchain hydrogen bonds are found in the region near the C-termini of pVI as indicated in the right panel.

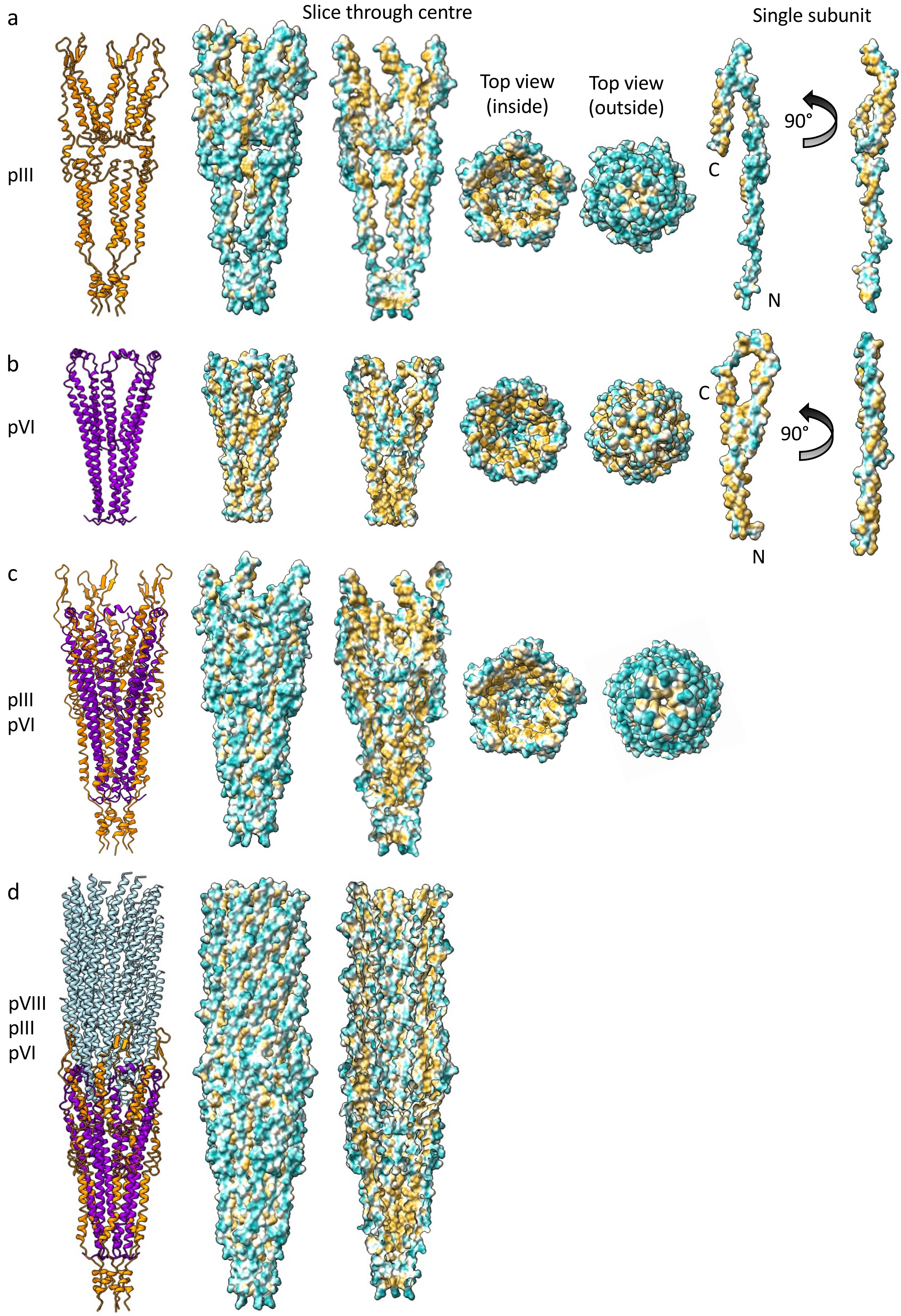

Supplementary Figure 6. Hydrophobicity of the pointy tip.

Surfaces are coloured by hydrophobicity with the most hydrophilic residues shown in cyan and the most hydrophobic in mustard yellow.

(a) pIII bundle in side view shown as ribbons, and as surfaces in side view, side view sliced through the centre to show the inside of the bundle, top view inside, top view outside. A single chain of pIII is shown in two views 90° apart. Density for the pIII N1-N2 domains and glycine linkers, plus the final 2 residues at the C-terminus, is not visible in our map and is not shown.
(b) pVI bundle in side view shown as ribbons, and as surfaces in side view, side view sliced through the centre to show the inside of the bundle, top view inside, top view outside. A single chain of pVI is shown in two views 90° apart.
(c) pIII / pVI bundle in side view shown as ribbons, and as surfaces in side view, side view sliced through the centre to show the inside of the bundle, top view inside, top view outside.
(d) pIII / pVI / pVIII bundle in side view shown as ribbons, and as surfaces in side view and side view sliced through the centre to show the inside of the bundle. Density for the surface exposed 4 residue sequence at the N-terminus of pVIII (AEGD, overall hydrophilic, see Supplementary Fig. 9) is not visible in our map and is not shown.

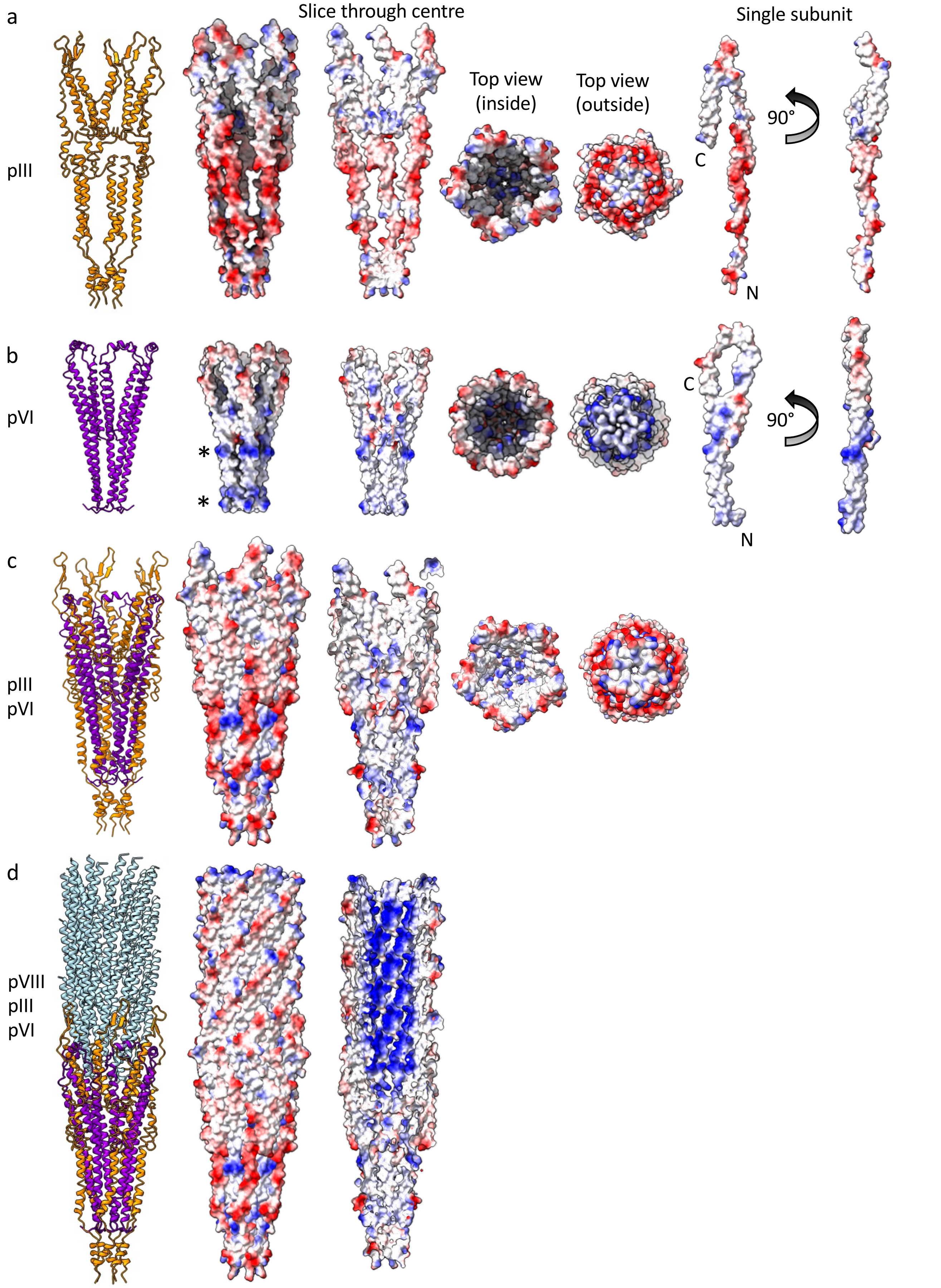

Supplementary Figure 7. Electrostatic potential of the pointy tip.

Surfaces are coloured by electrostatic potential with negative residues shown in red and positive residues in blue.

(a) pIII bundle in side view shown as ribbons, and as surfaces in side view, side view sliced through the centre to show the inside of the bundle, top view inside, top view outside. A single chain of pIII is shown in two views 90° apart. Density for the pIII N1-N2 domains and glycine linkers, plus the final 2 residues at the C-terminus, is not visible in our map and is not shown.

(b) pVI bundle in side view shown as ribbons, and as surfaces in side view, side view sliced through the centre to show the inside of the bundle, top view inside, top view outside. A single chain of pVI is shown in two views 90° apart. * denotes distinct rings of positive charge.

(c) pIII / pVI bundle in side view shown as ribbons, and as surfaces in side view and side view sliced through the centre to show the inside of the bundle, top view inside, top view outside.

(d) pIII / pVI / pVIII bundle in side view shown as ribbons, and surfaces in side view and side view sliced through the centre to show the inside of the bundle. Density for the surface exposed 4 residue sequence at the N-terminus of pVIII (AEGD, overall negatively charged, see Supplementary Fig. 10) is not visible in our map and is not shown.

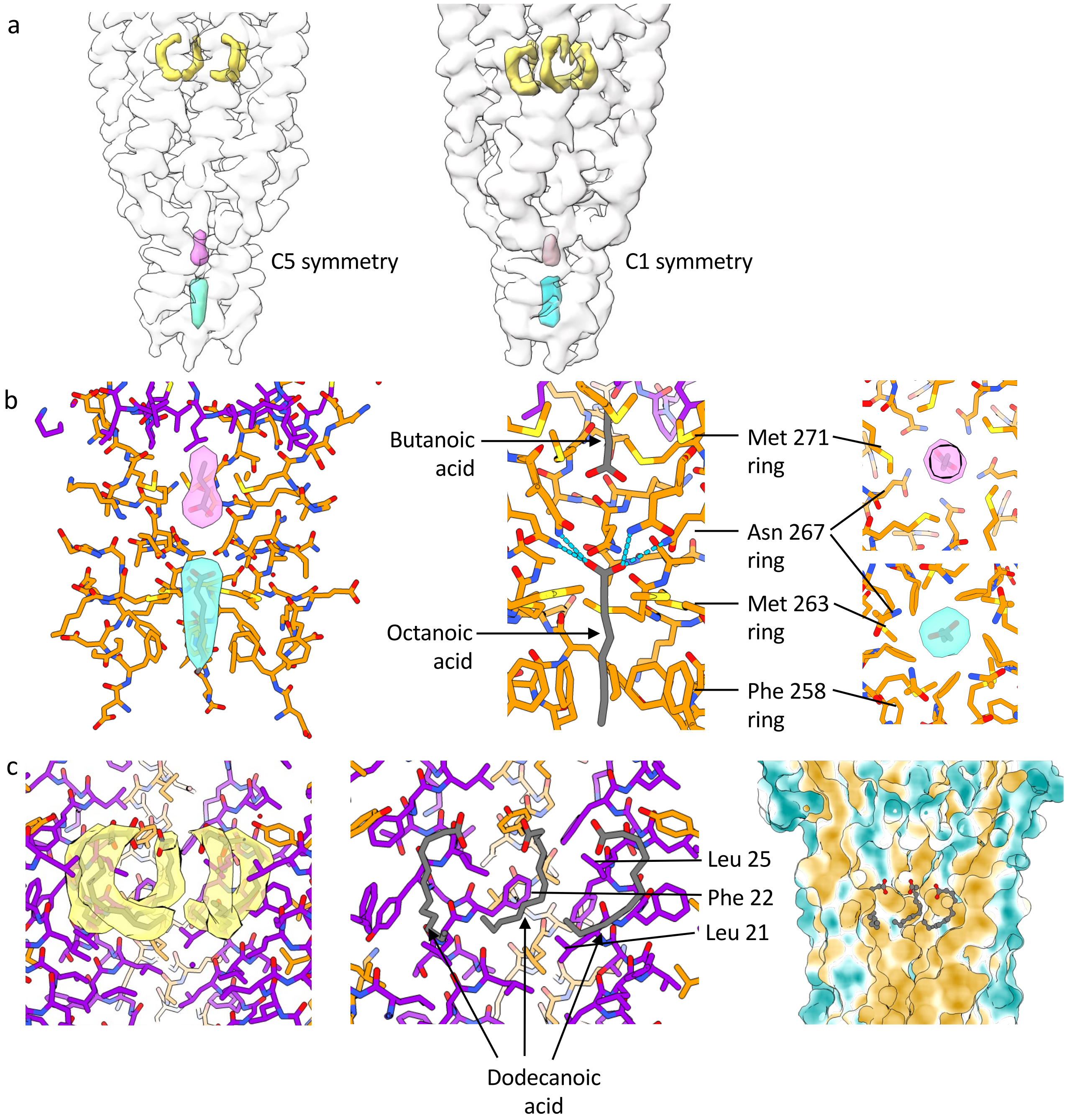

Supplementary Figure 8. Fatty acid molecules at the pointy tip.

(a) Three areas of unaccounted-for density were observed in the central lumen of the pointy tip (coloured cyan, pink and yellow). The three densities were also observed in a map of the pointy tip which was determined without symmetry applied to rule out symmetry-related artefacts.

(b) The binding pockets at the tip for the cyan and pink densities are mainly hydrophobic with some hydrogen-bonding possibilities. Therefore, we modelled in fatty acids octanoic acid (cyan density) and butanoic acid (pink density). Hydrogen bonds are shown as blue dashed lines. The binding pocket for the cyan density is composed of rings of phenylalanine, methionine and asparagine residues. The pink density shares the ring of asparagine residues and has an additional ring of methionines.

(c) 5 symmetry-related tubes of density observed at the pointy tip had the appearance of fatty acids and are located in a hydrophobic pocket. These have been modelled in as dodecanoic acid (yellow density). The pocket is lined with

the hydrophobic residues Leu 21, Phe 22 and Leu 25. Right hand panel shows the phage coloured by hydrophobicity with hydrophilic residues shown in cyan and hydrophobic residues in mustard yellow.

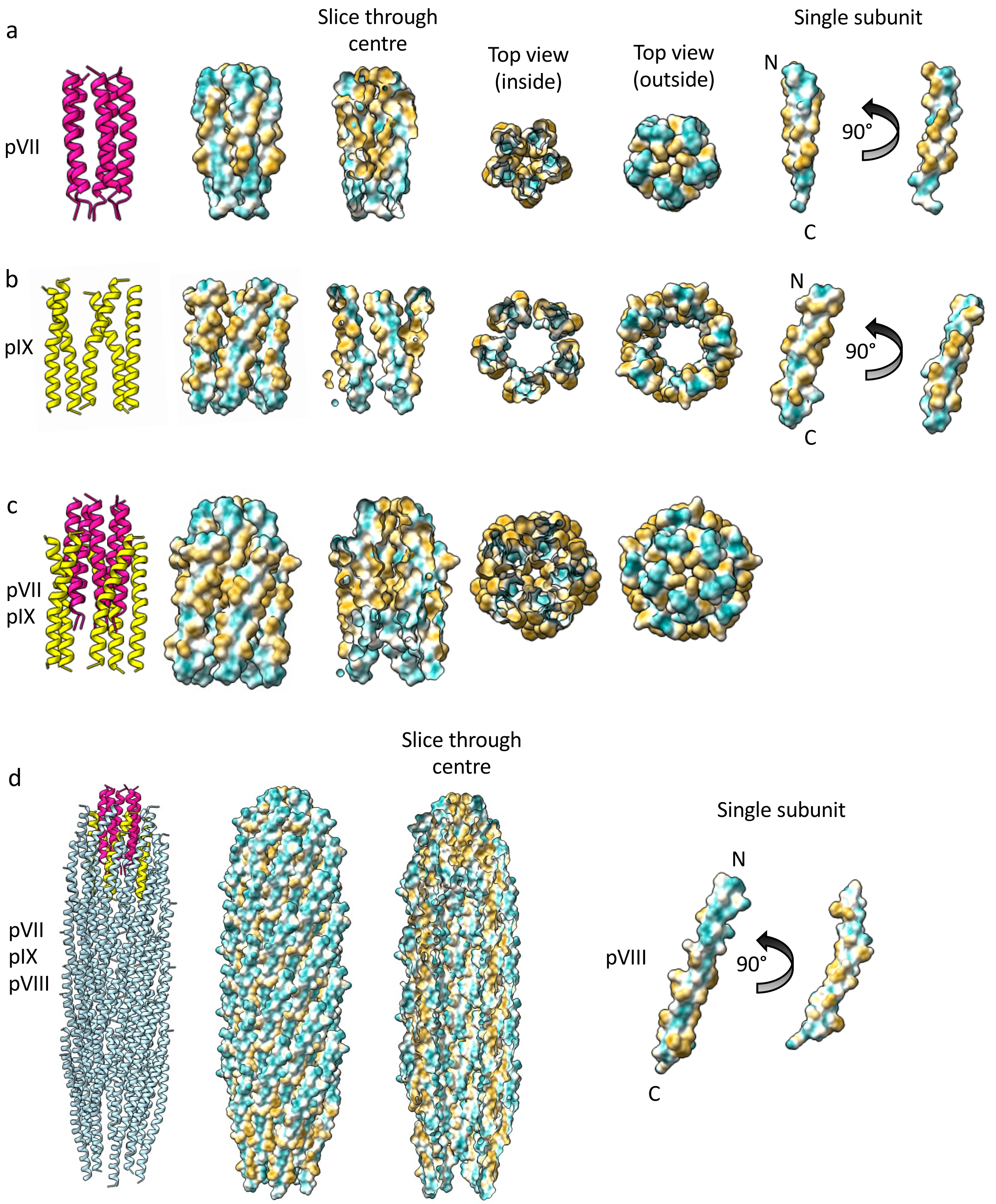

Supplementary Figure 9. Hydrophobicity of the round tip.

Surfaces are coloured by hydrophobicity with the most hydrophilic residues shown in cyan and the most hydrophobic in mustard yellow.

(a) pVII bundle in side view shown as ribbons, and as surfaces in side view, side view sliced through the centre to show the inside of the bundle, top view inside, top view outside. A single chain of pVII is shown in two views 90° apart. Density for the surface exposed 5 residue sequence at the N-terminus of pVII (MEQVA, of mixed hydrophobicity) is not visible in our map and is not shown.

(b) pIX bundle in side view shown as ribbons, and as surfaces in side view, side view sliced through the centre to show the inside of the bundle, top view inside, top view outside. A single chain of pIX is shown in two views 90° apart.

(c) pVII / pIX bundle in side view shown as ribbons, and as surfaces in side view, side view sliced through the centre to show the inside of the bundle, top view inside, top view outside.

(d) pVII / pIX / pVIII bundle in side view shown as ribbons, and as surfaces in side view and side view sliced through the centre to show the inside of the bundle. A single chain of pVIII is shown in two views 90° apart. Density for the surface exposed 4 residue sequence at the N-terminus of pVIII (AEGD, overall hydrophilic) is not visible in our map and is not shown.

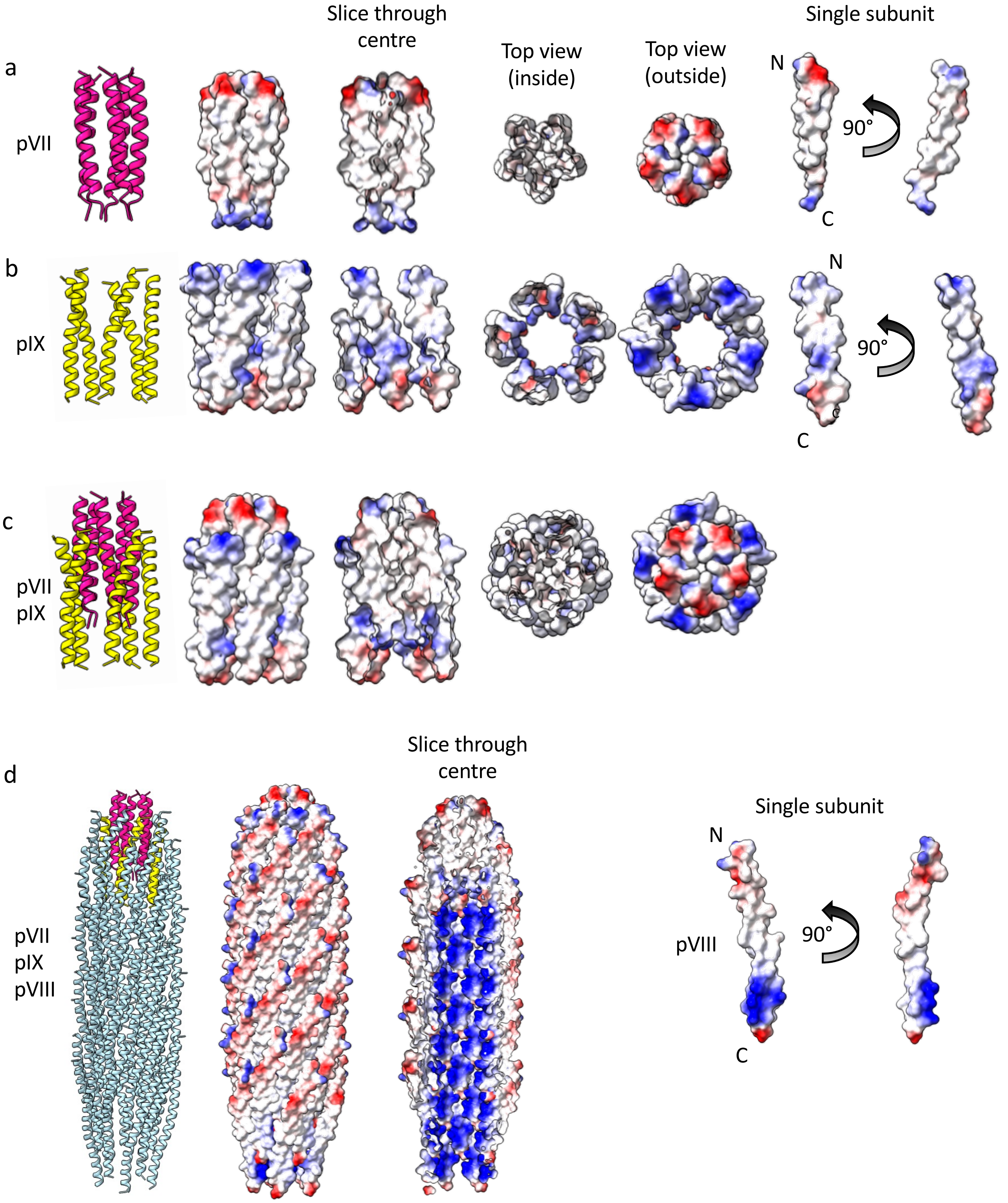

Supplementary Figure 10. Electrostatic potential of the round tip.

Surfaces are coloured by electrostatic potential with negative residues shown in red and positive residues in blue.

(a) pVII bundle in side view shown as ribbons, and as surfaces in side view, side view sliced through the centre to show the inside of the bundle, top view inside, top view outside. A single chain of pVII is shown in two views 90° apart. Density for the surface exposed 5 residue sequence at the far N-terminus of pVII is not visible in our map and is not shown. This sequence (MEQVA) would contribute to the negative charge at the round tip.

(b) pIX bundle in side view shown as ribbons, and as surfaces in side view, side view sliced through the centre to

show the inside of the bundle, top view inside, top view outside. A single chain of pIX is shown in two views 90° apart.

(c) pVII / pIX bundle in side view shown as ribbons, and as surfaces in side view, side view sliced through the centre to show the inside of the bundle, top view inside, top view outside.

(d) pVII / pIX / pVIII bundle in side view shown as ribbons, and surfaces in side view and side view sliced through the centre to show the inside of the bundle. A single chain of pVIII is shown in two views 90° apart. Density for the surface exposed 4 residue sequence at the N-terminus of pVIII (AEGD, overall negatively charged) is not visible in our map and is not shown.

a

```
f1     AEGD---DPAKAAFDSLQASATEYIGYAWAMVVVIVGATIGIKLFKKFTSKAS
fd     AEGD---DPAKAAFDSLQASATEYIGYAWAMVVVIVGATIGIKLFKKFTSKAS
M13    AEGD---DPAKAAFNSLQASATEYIGYAWAMVVVIVGATIGIKLFKKFTSKAS
Ike    AEPNAATNYATEAMDSLKTQAIDLISQTWPVVTTVVVAGLVIRLFKKFSSKAV
       ** :   : *. *::**::.* : *. :*.:*..:* * : *:*****:***
```

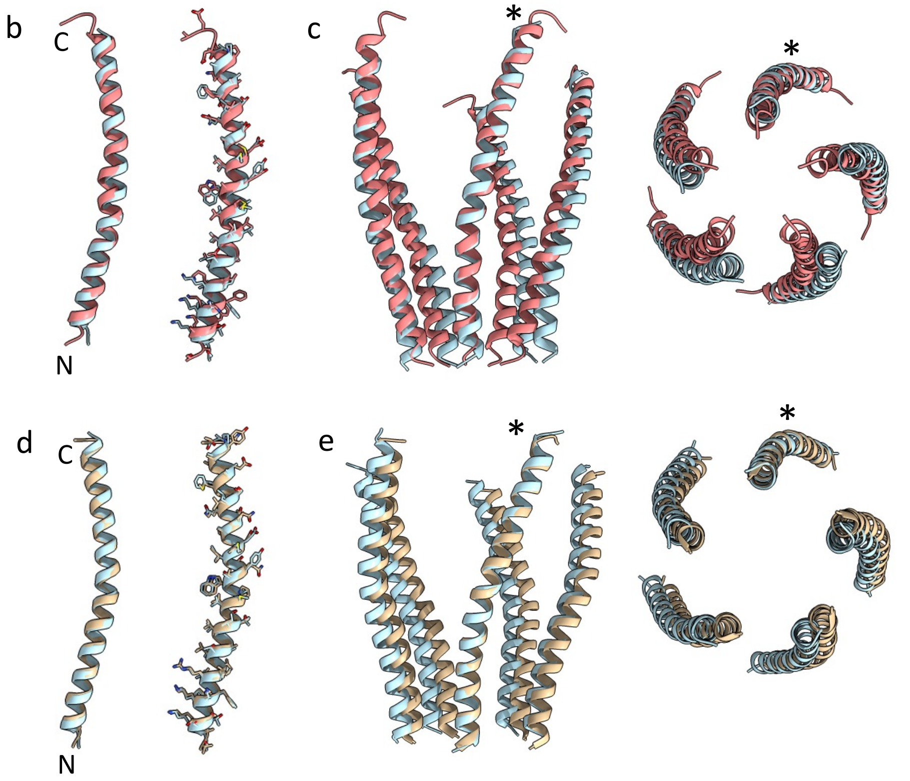

Supplementary Figure 11. Comparison of the pVIII structure to other phage filaments.

(a) Sequence alignment of pVIII from f1, fd, M13 and Ike calculated with T-Coffee[5]. The signal sequences were removed for the alignment. The mature form of pVIII from f1 is 100 % identical to that from fd, 98 % identical to that from M13 (1 amino acid different) and 40 % identical to Ike. Conserved residues are labelled with *, strongly similar with : and weakly similar with .

(b) Overlay of the f1-derived nanorod pVIII monomer (light blue) with a monomer of pVIII from fd (2C0W[6], structure determined by X-ray fibre diffraction, coral) in cartoon and showing sidechains.

(c) Overlay of the nanorod pentamer on the fd (2C0W) pentamer (by aligning one monomer from the nanorod with one monomer from 2C0W) in side and top views. The helices which were aligned are marked with *.

(d) Overlay of the nanorod pVIII monomer (light blue) with the Ike monomer (6A7F[7], structure determined by cryoEM, tan) in cartoon and showing sidechains.

(e) Overlay of the nanorod pentamer on the Ike pentamer (by aligning one monomer from the nanorod with one monomer from 6A7F) in side and top views. The helices which were aligned are marked with *.

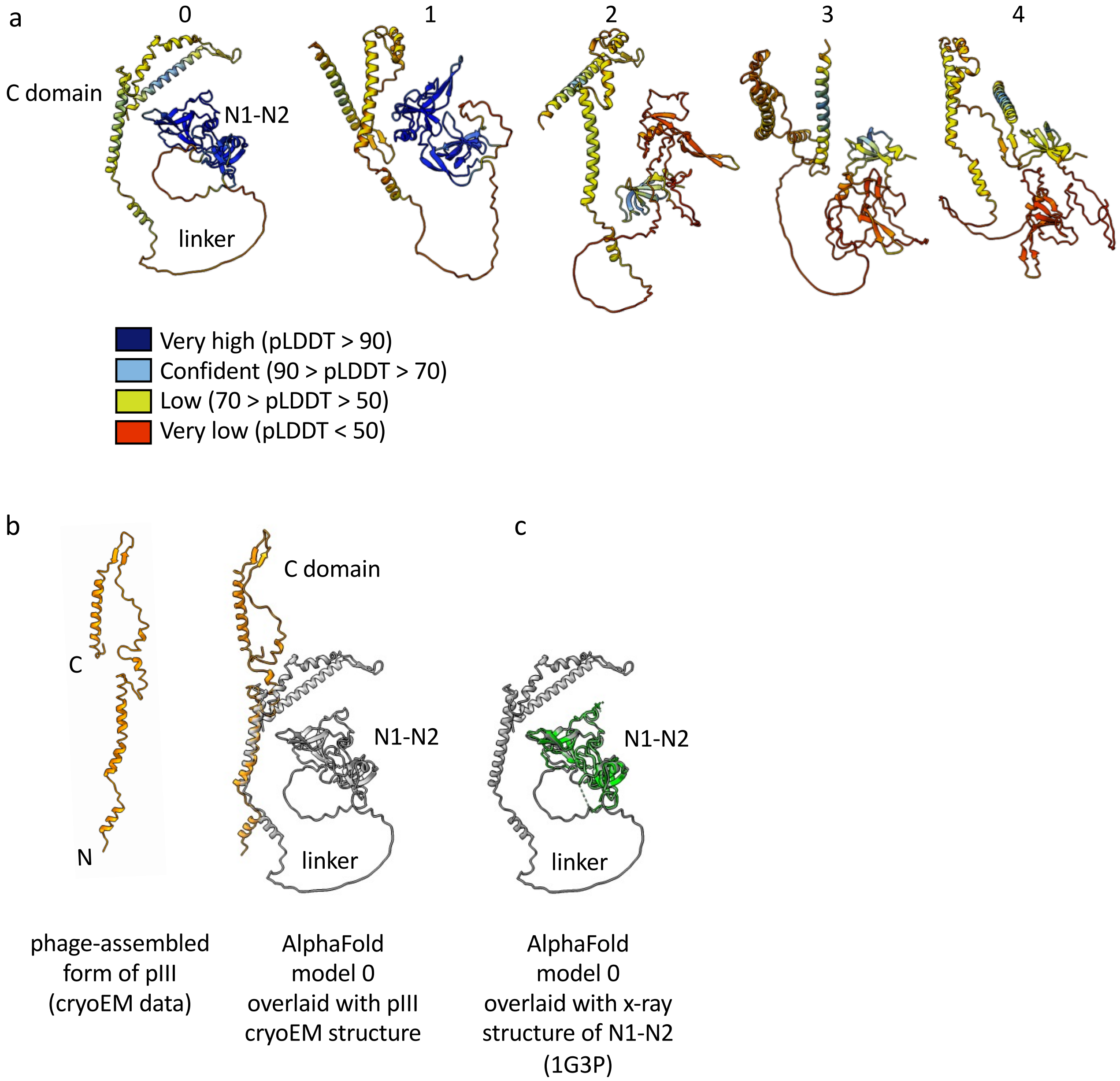

Supplementary Figure 12. AlphaFold predictions of the pIII N1-N2 domains and linkers.

(a) AlphaFold predictions of the full-length pIII monomer coloured by confidence levels with highest confidence in blue, through cornflower blue, then yellow to the lowest confidence in dark orange. Numbers relate to the confidence ranking from AlphaFold (0 is best, 4 is worst). The N1-N2, linker and C domains are labelled in model 0. The N1-N2 domains are predicted at high confidence (based on X-ray structures 1G3P[8] and 2G3P[9]), the C domain at low confidence, and the linker at very low confidence. A pLDDT score of <50 is indicative of disorder in the linker region[10].

(b) The flexible linker between the C domain and N1-N2 domains likely allow the receptor binding domains to exist in a continuum of different conformations relative to the pointy tip of the phage. To make a composite model of the f1-derived nanorod, we used the top prediction of pIII from AlphaFold (model 0 in (a), grey) and overlaid it with our structure of the C domain of pIII (orange) to obtain relative positions of the N1-N2 domains. The structure of the pIII C domain in the phage-assembled structure is shown for comparison.

(c) Top prediction of pIII from AlphaFold (model 0 in (a)) shown in grey overlaid with the crystal structure of the N1-

N2 domains of pIII (1G3P)[8] shown in green to demonstrate a good fit (RMSD 0.8 Å between 185 pruned atom pairs). The N1-N2 and linker domain prediction was subsequently modelled onto the tip of the C domain structure to generate the model of complete pIII in the assembled phage.

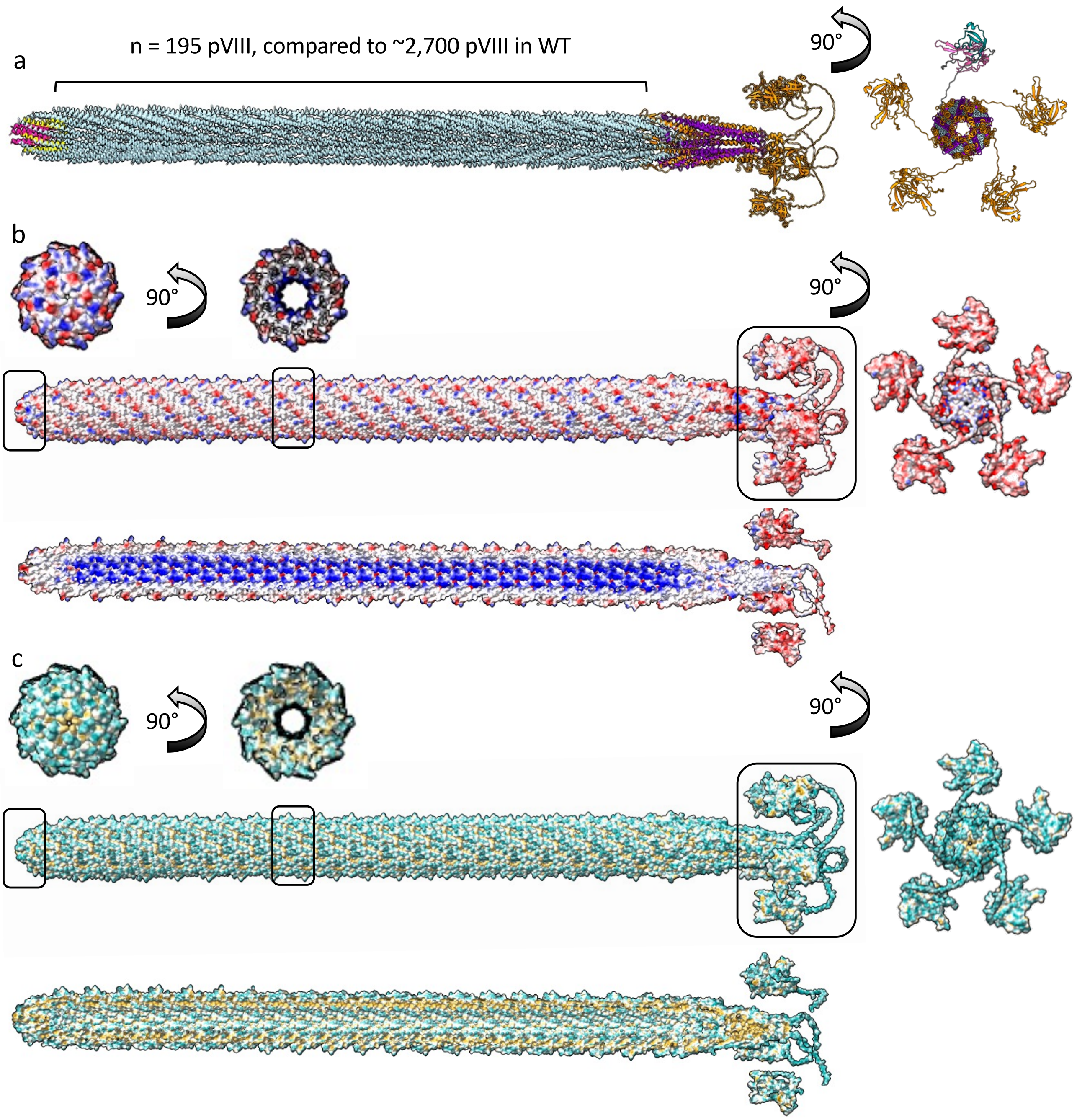

Supplementary Figure 13. Composite model of the f1-derived nanorod.

(a) A composite model was generated by aligning the pointy and round tip structures with 195 copies of the pVIII protein from the filament structure, and an AlphaFold model of the linkers and N1-N2 domains of pIII (model 0 in Supplementary Fig. 12a). WT phage contains ~2,700 copies of pVIII and would be ~12x longer than the structure shown. pVII is shown in pink, pIX in yellow, pIII in orange, pVI in purple and pVIII in light blue. Right, view of the pointy tip with four of the N-terminal regions of pIII shown in orange, with one coloured differently to highlight the N1 domain (teal), N2 domain (light pink) and flexible linker regions (grey).

(b) f1-derived nanorod shown in surface representation and coloured by electrostatic potential. The outer surface and a view sliced through the centre are shown, with negative residues coloured in red and positive residues in blue. Boxed areas show views rotated by 90°.

(c) f1-derived nanorod shown in surface representation and coloured by hydrophobicity. The outer surface and a

view sliced through the centre are shown, with the most hydrophilic residues coloured in cyan and the most hydrophobic in mustard yellow. Boxed areas show views rotated by 90°.

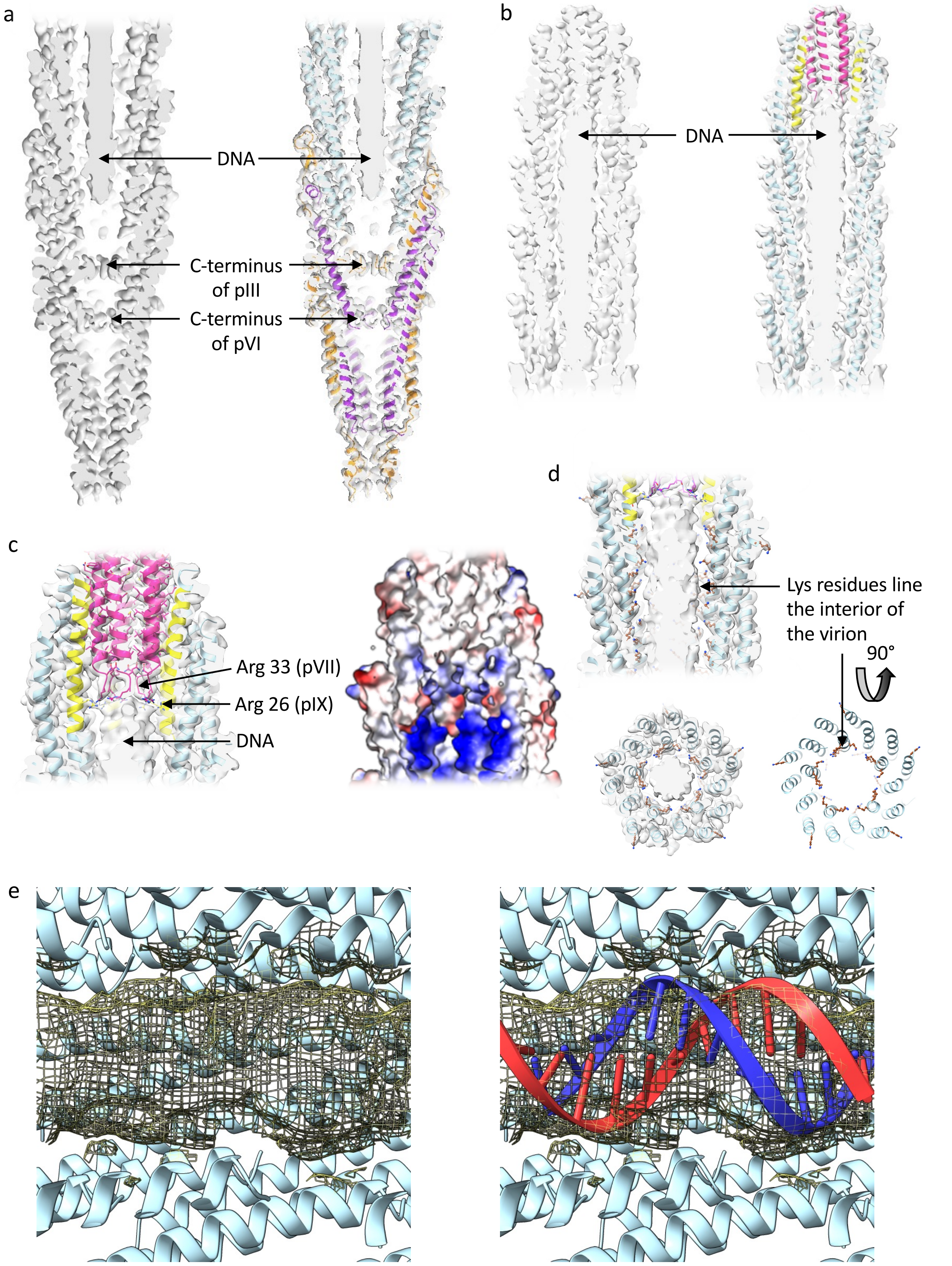

Supplementary Figure 14. Phage DNA.

(a) Slice through the centre of the pointy tip with density map shown in light grey and protein chains as cartoon. pIII is shown in orange, pVI in purple and pVIII in light blue. The density for the DNA is visible in the centre.

(b) A slice through the centre of the round tip with density map shown in light grey and protein chains as cartoon. pVII is shown in pink, pIX in yellow and pVIII in light blue. The density for the DNA is clearly visible in the centre and butts up to the round tip.

(c) Sidechains Arg 33 of pVII and Arg 26 of pIX are shown as sticks, forming a highly positively charged pocket for the DNA packaging signal hairpin to bind.

(d) Lysine residues of protein pVIII are shown as brown sticks. Four lysine residues at the C-terminal end of pVIII line the virion cavity, causing it to be positively charged. Shown in side and top views 90° apart.

(e) Density at the centre of the phage displayed around the central axis with a 12mer of B-DNA modelled (individual strands coloured red and blue).

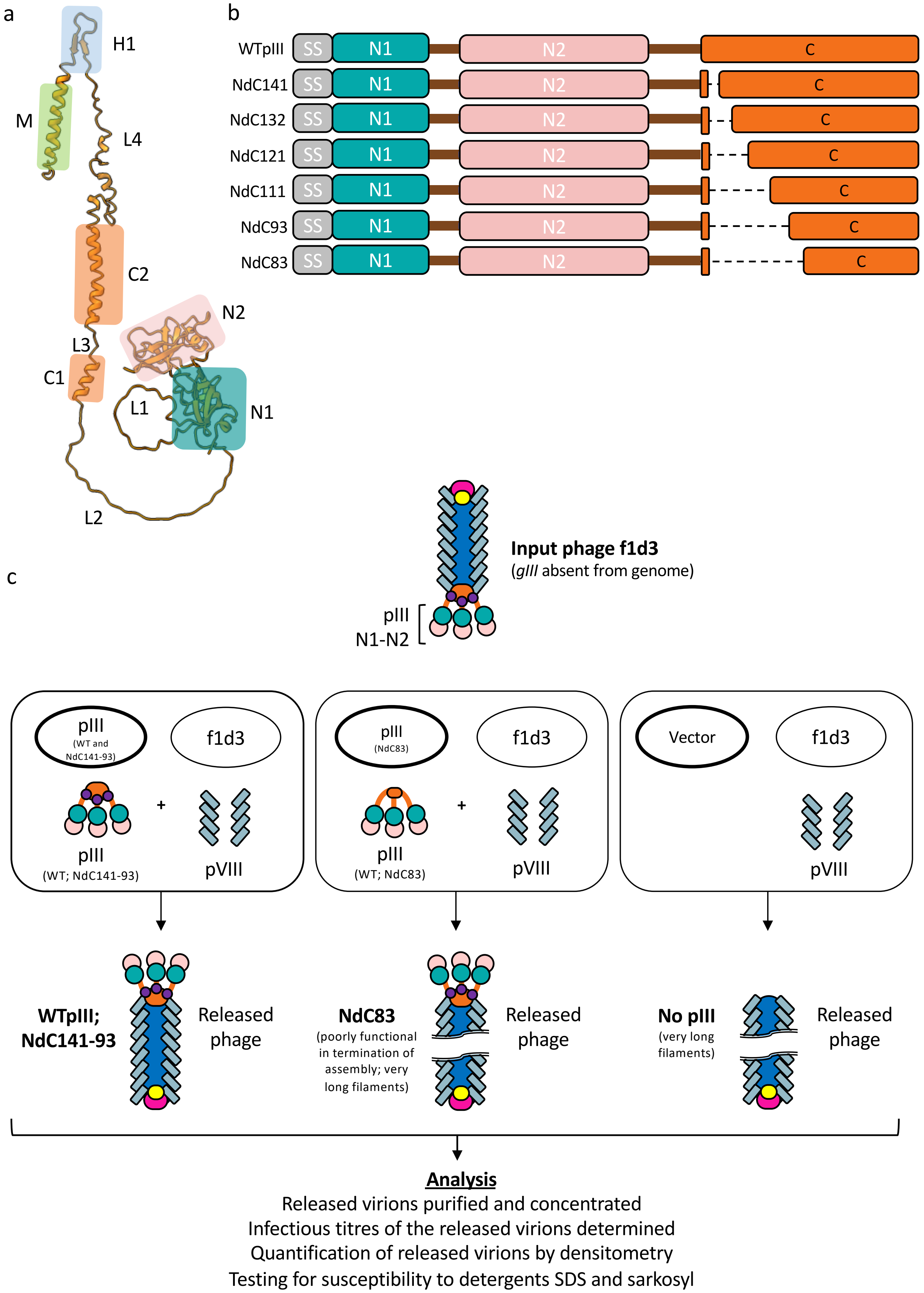

Supplementary Figure 15. Production of f1d3 phage containing C domain truncations.

(a) Domain organisation of pIII. From the N-terminus of the protein: domain N1 (N1), glycine rich linker 1 (L1), domain N2 (N2), glycine-rich linker 2 (L2), C-terminal domain 1 (C1), linker 3 (L3), C-terminal domain 2 (C2), linker

4 (L4), β-hairpin loop (H1) and transmembrane helix (M).

(b) Schematic to show domain organisation of wild type pIII and nested deletion (Nd) mutants (NdC141, NdC132, NdC121, NdC111, NdC93, NdC83)[11]. SS, signal sequence (grey); N1, N1 domain (teal); N2, N2 domain (light pink); C, C domain (orange). The glycine linkers are shown as dark brown boxes, and the deleted regions as dashed black lines.

(c) Diagram showing experiment for production of phages containing nested deletion mutant proteins. For clarity only 3 pIII proteins, instead of 5, are shown.

Top: input f1d3 phage stock carrying a complete deletion of *gIII* from the genome. Full-length pIII in the phage coat is supplied *in trans* from a plasmid. This stock was produced from *E. coli* strain K1976, expressing WT pIII from plasmid pJARA112[12]. The major capsid protein pVIII is shown in light blue, the minor capsid proteins pVII / pIX in pink and yellow respectively, and the minor capsid protein pVI in purple. The C domain of the minor capsid protein pIII is shown in orange, with the N1 and N2 domains of pIII in teal and light pink respectively, as per panel (b).

Boxed panels: host cell plasmid (bold oval) and phage combination (fine oval) used for nanorod production, and the expected composite phage particle shown underneath.

Left and centre box: WTpIII and NdC141-NdC83 mutants are expressed from plasmid pJARA200, under the control of a *lacUV5* promoter[11]. NdC83 is shown separately (middle box), as most of the phage particles remain attached to the host cells, with a small fraction released into the media as very long multi-length filaments, as per the negative control[13] (right box).

Right box: non-complemented f1d3 infection (using vector pBR322 that does not supply pIII).

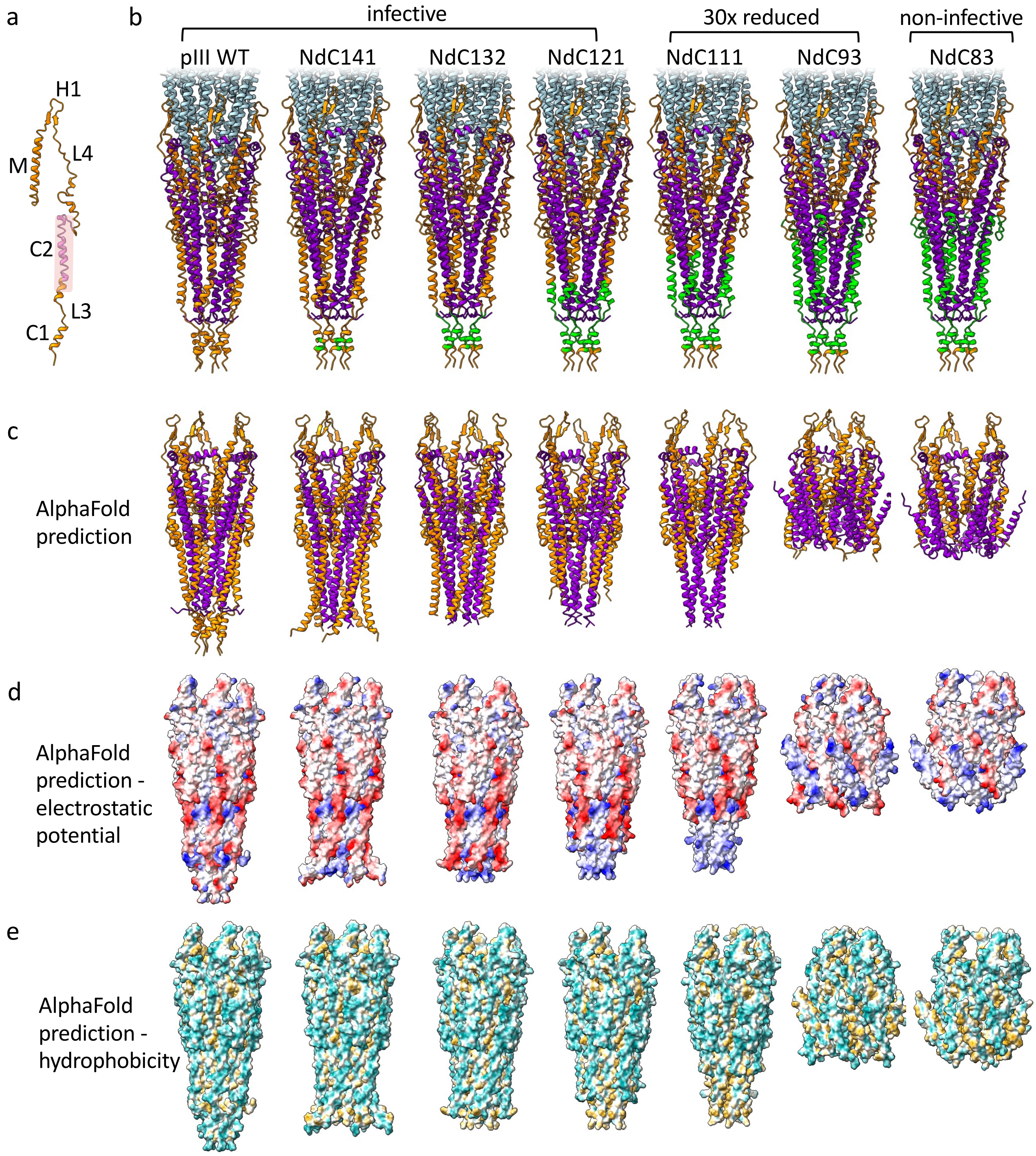

Supplementary Figure 16. Functional analysis of the C domain in infection.

(a) Ribbon diagram of pIII domains, from the N-terminus of the C domain: C-terminal domain 1 (C1), linker 3 (L3), C-terminal domain 2 (C2), linker 4 (L4), β-hairpin loop (H1) and transmembrane helix (M). The N1 and N2 domains and glycine-rich linkers are not shown. The infection-competence segment (ICS) is boxed in pink.

(b) C-terminal deletion mutants are mapped onto the pointy tip structure in green - from left to right: wild type, NdC141, NdC132, NdC121, NdC111, NdC93 and NdC83.

(c) AlphaFold predictions of the mutation series shown as ribbons. pIII is shown in orange and pVI in purple.

(d) AlphaFold predictions of the mutation series shown in surface representation and coloured by electrostatic potential. Negative residues are shown in red and positive residues in blue.

(e) AlphaFold predictions of the mutation series shown as surface representation coloured by hydrophobicity. The most hydrophilic residues are coloured in cyan and the most hydrophobic in mustard yellow. As more pIII is removed, more of the hydrophobic helices of pVI are exposed, which begin to fold up in the mutants with the largest deletions.

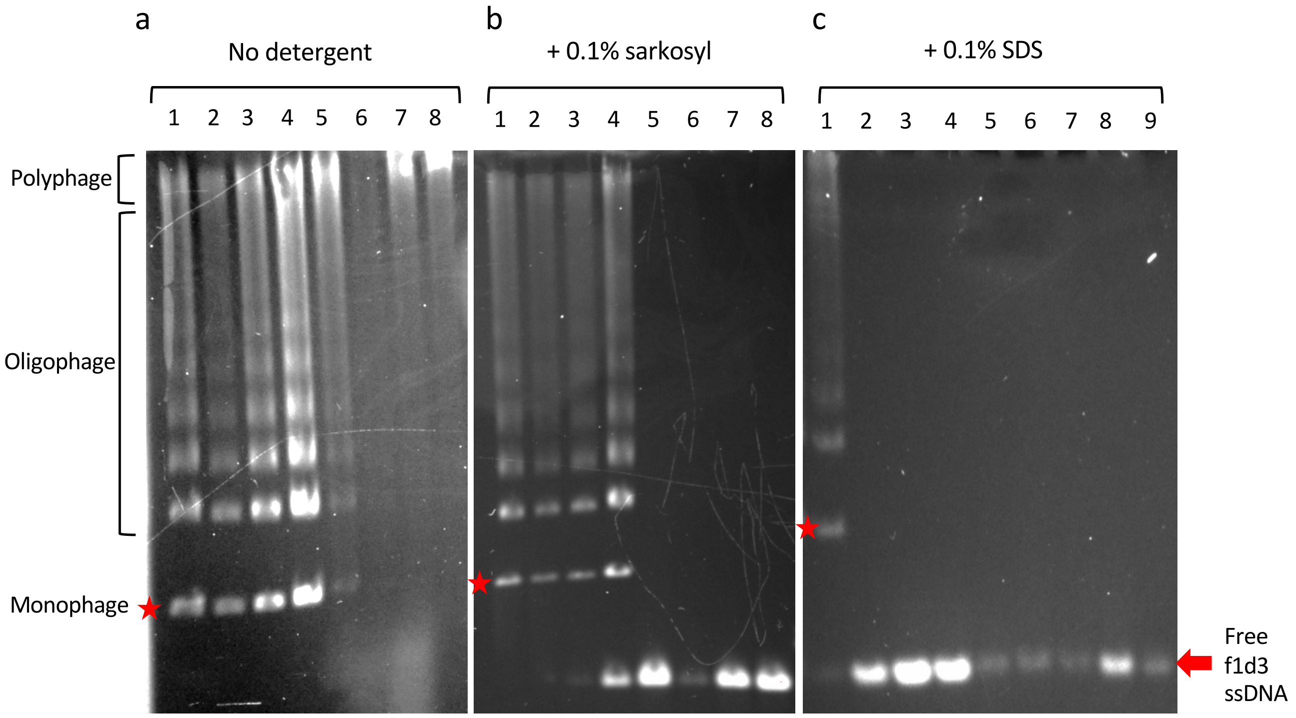

Supplementary Figure 17. Native agarose gel electrophoresis to test the stability of phages to detergents with different properties.

Panels show migration of phages and released (free) phage ssDNA in agarose gels (a) without or (b) with pre-incubation in 0.1% sarkosyl or (c) in 0.1% SDS. The position of the virions in the gel is only visible after *in situ* disassembly by soaking the gel in an alkaline solution (see Supplementary Methods).

Numbered lanes: Phages containing (1) WT pIII; (2) NdC141; (3) NdC132; (4) NdC121; (5) NdC111; (6) NdC93; (7) NdC83; (8) no pIII; (9) purified f1d3 ssDNA.

Purified f1d3 ssDNA (9) serves as a standard for DNA released from the phage by detergent treatment; commercial circular single-stranded standards are not available. Phage containing WT pIII serves as a standard for the native virions given that they migrate differently from any available DNA standards[14,15]. The f1d3 phage complemented by pIII expressed *in trans* have a wide distribution of length[15]. Monophages (red asterisk) are phage particles containing a single f1d3 genome. Oligophages are phage particles containing two to several sequentially packaged f1d3 genomes. Polyphages are multiple-length phage particles containing sequentially packaged f1d3 genomes. These virions form more diffuse bands than the monomer or free ssDNA[11,15].

Free f1d3 DNA (red block arrow) is ssDNA released from the phage during pre-incubation with detergents, or purified f1d3 ssDNA used as a standard. The analysis was performed in triplicate.

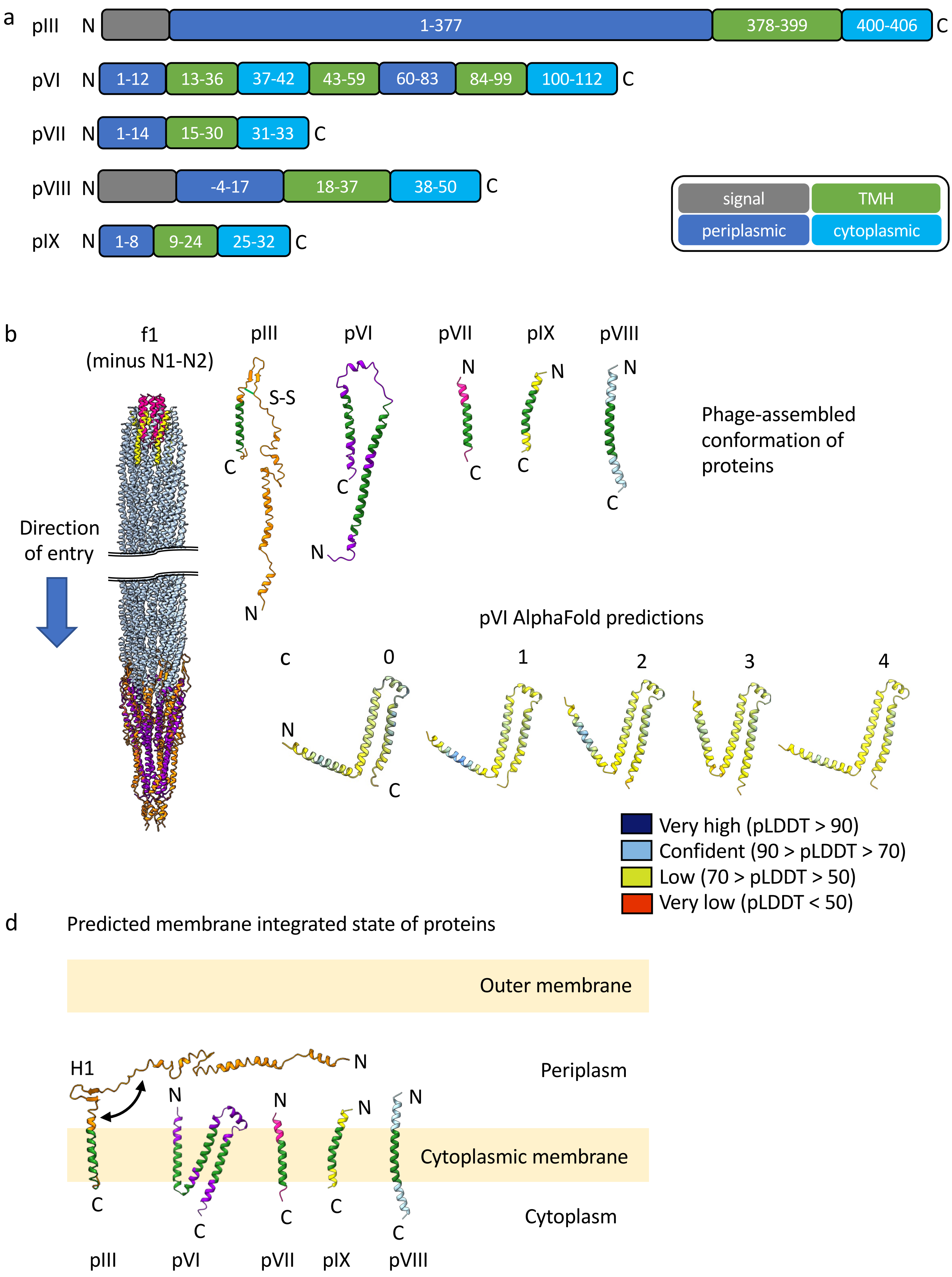

Supplementary Figure 18. Membrane integration of phage capsid proteins.

(a) Transmembrane region predictions using MEMSAT-SVM[16] via the PSIPRED server[17]. Signal sequences are shown in grey, transmembrane helices (TMH) in green, periplasmic regions in dark blue and cytoplasmic regions in bright blue. The numbering of pIII and pVIII is for the mature proteins (excluding their signal sequences). The signal

sequence predicted for pVIII varies slightly from the Uniprot entry which shows the signal sequence to consist of residues 1-23, hence the negative numbering for the periplasmic domain.

(b) For clarity, a version of f1 phage without the N1-N2 domains of pIII is shown with the orientation of individual proteins in their phage-assembled conformations. The predicted transmembrane regions are coloured green and the disulphide bond in pIII is shown in green and labelled S-S.

(c) AlphaFold predictions of the pVI protein coloured by confidence levels with highest confidence in blue, through cornflower blue, then yellow to the lowest confidence in dark orange. Numbers relate to the confidence ranking from AlphaFold (0 is best, 4 is worst). The N and C-termini are labelled in model 0. Interestingly, AlphaFold predicts that the long N-terminal helix of pVI seen in the phage-assembled structure (b) is folded up into two smaller helices, in agreement with the transmembrane prediction in (a).

(d) Orientation of phage proteins in the cytoplasmic membrane according to MEMSAT-SVM prediction. The pIII conformation was modelled by swinging the N-terminal part of the C domain out around the β-hairpin loop (H1) according to the arrow. The pVI conformation is from AlphaFold model 3. Proteins are coloured as per panel (b) with the N and C-termini labelled.

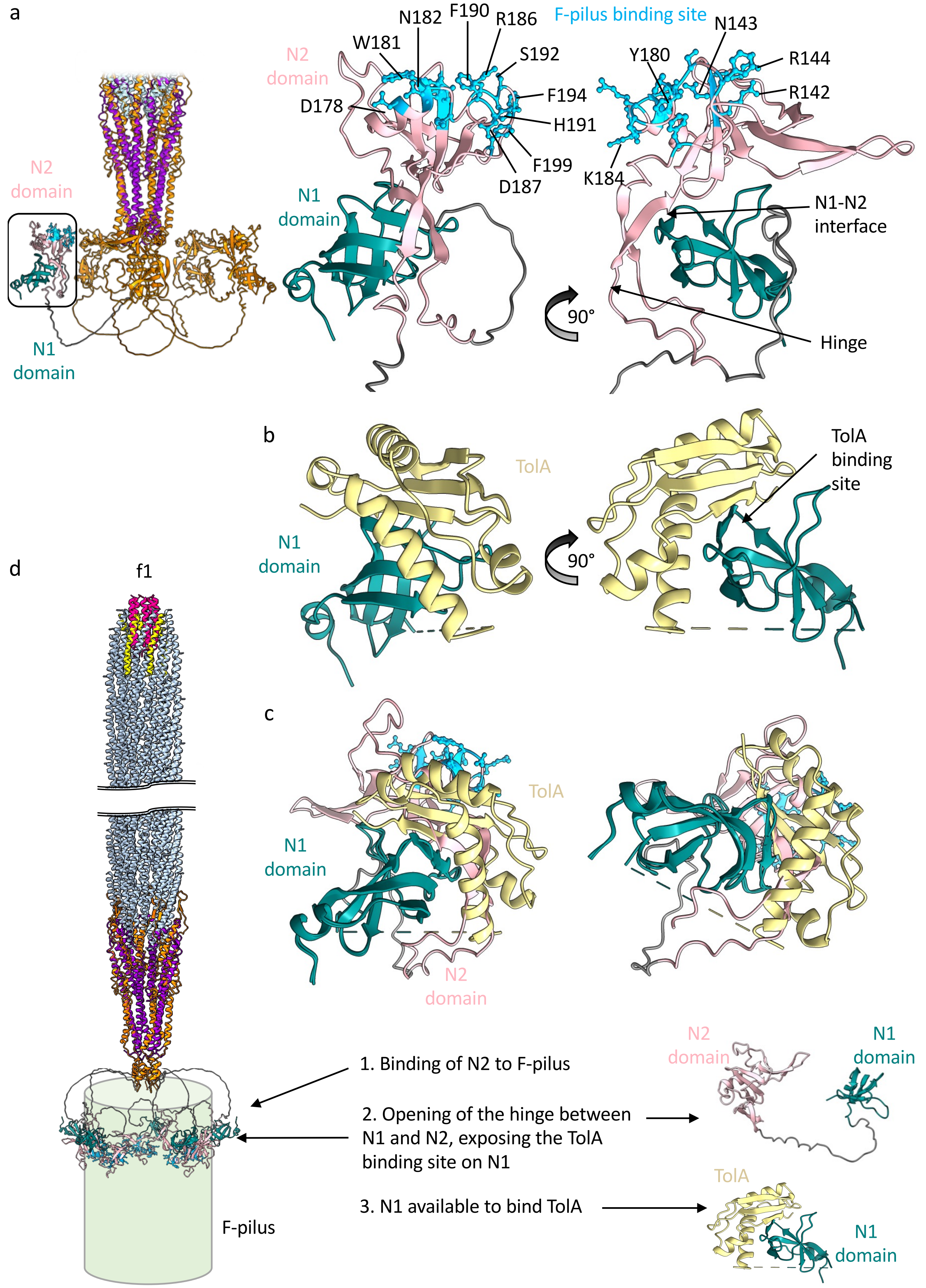

Supplementary Figure 19. The F-pilus and TolA binding sites on pIII.

(a) Overview of the pointy tip to show the N1-N2 domains attached to the C domain of pIII via flexible linkers, based

on PDB 1G3P[8]. N1 is shown in teal, N2 in light pink, with the residues that make up the F-pilus binding site on N2 shown as cyan ball-and-stick. The boxed region is shown in detail in the following panels in two views 90° apart.

(b) The N1 domain (teal) complexed with TolA (yellow), shown with N1 in the same orientations as in part (a), based on PDB 1TOL[18].

(c) Overlay of the N1-N2 structure and N1-TolA complex in 2 views (overlaid on the N1 domains) to show that N2 and TolA share the same binding site on the N1 domain.

(d) The N1-N2 domains of pIII could bind to a point on the F-pilus due to flexibility in the glycine-rich linkers between N1-N2 and the C domain of pIII. The N1-N2 domains were manually swung down relative to the C domain of pIII via the flexible linkers to model the position of the N1-N2 domains on F-pilus binding. 5 copies of N2 are shown bound to the F-pilus, but it is also possible that infection occurs after a single N2 binding event. Binding of N2 to the F-pilus likely causes a conformational change in the N1-N2 domains, displacing N1 from N2 by movement of the hinge region between the two, and consequently exposing the TolA binding site on N1. The open N1-N2 structure was made by manually opening the flexible hinge region in PBD 1G3P[8], guided by the position of N1 bound to TolA in PDB 1TOL[18].

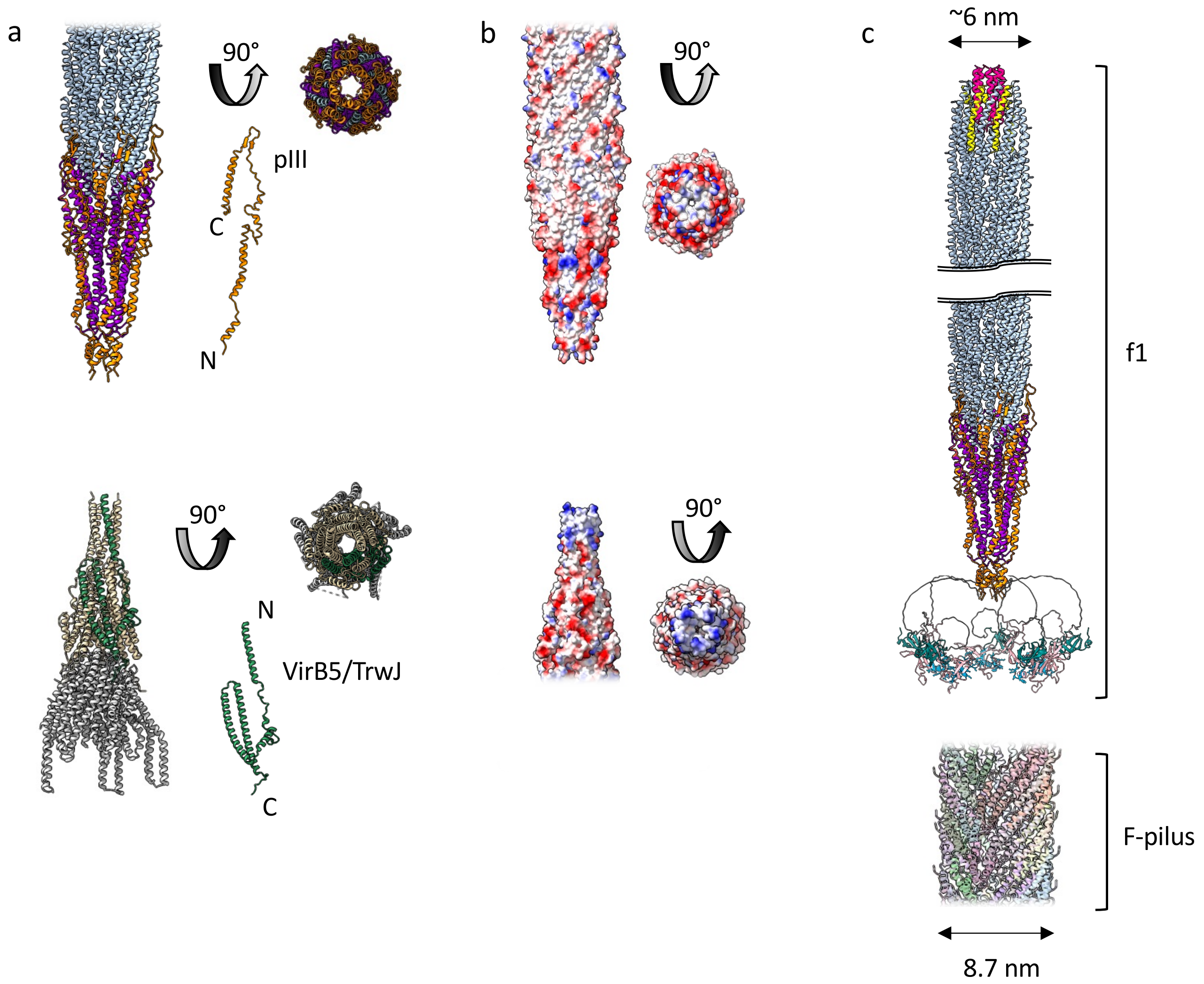

Supplementary Figure 20. f1 binding to the F-pilus.

(a) The f1-derived nanorod pointy tip and the VirB5/TrwJ protein (part of the type IV secretion system stalk; PDB 7O3V[19]) drawn to scale as cartoons, in side and top views. pIII at the pointy tip is shown in orange and VirB5/TrwJ in wheat, with one subunit coloured green. The remainder of the type IV secretion system stalk is coloured in charcoal. An individual chain of pIII is shown in orange and of VirB5/TrwJ in green.

(b) Surface representation of the f1 pointy tip and VirB5/TrwJ, with negative residues shown in red and positive residues in blue, shown in side and top views.

(c) f1 phage with N1-N2 domains drawn to scale with respect to the F-pilus (PDB 5LER)[20].

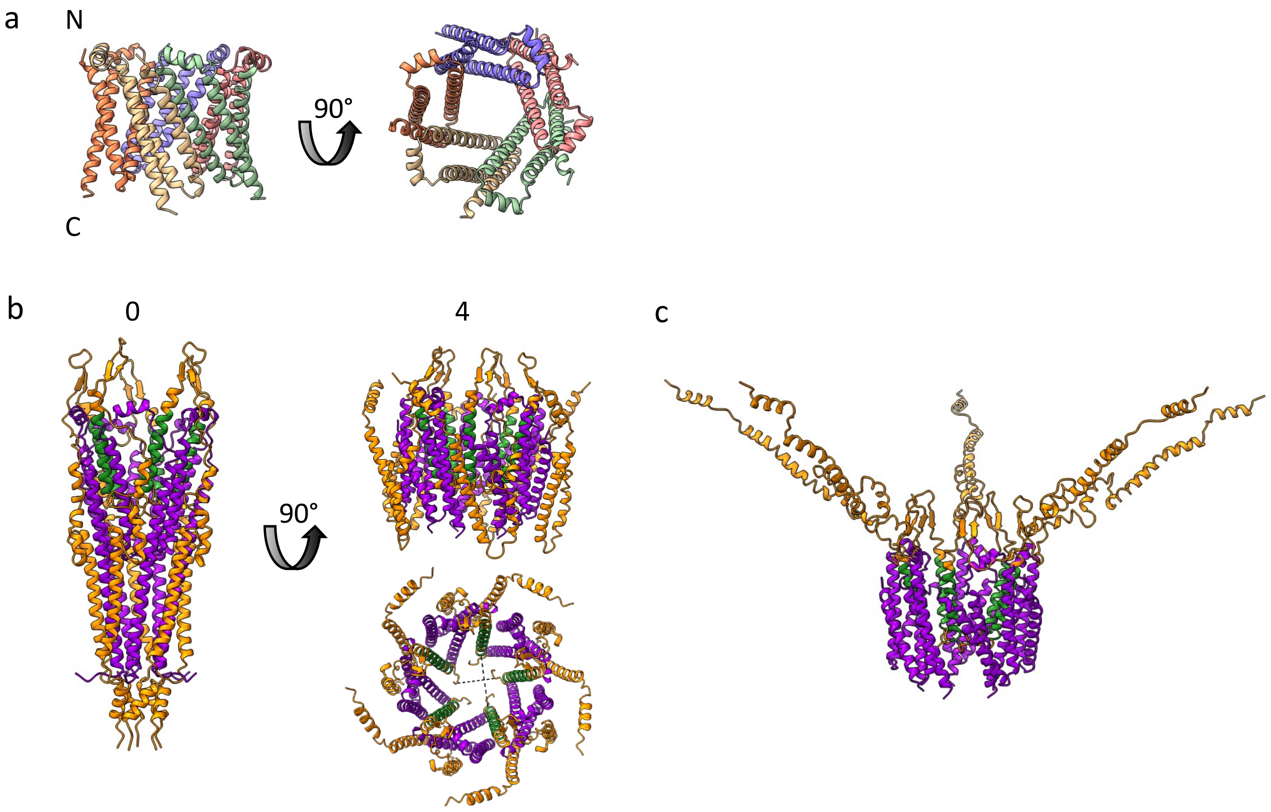

Supplementary Figure 21. AlphaFold predictions of the pVI pentamer, and pIII-pVI pentameric complex.

(a) Top AlphaFold prediction of the pVI pentamer coloured by chain in side and top views, depicting a pore.

(b) Top AlphaFold prediction (model 0) of a pentamer of the pIII C domain (orange) with a pVI pentamer (purple). This prediction is compared to the fifth AlphaFold prediction (model 4), the latter being more reminiscent of how the complex might look when embedded in the cytoplasmic membrane, with the proteins in a folded-up conformation. The predicted transmembrane helices of pIII are coloured green. The pointy end of the tip could peel back to allow insertion of the hydrophobic helices of pVI into the cytoplasmic membrane. A view shown looking down the channel reveals the dimensions of the pore. The diameter of the narrowest part of the open pore is between the C-terminal unstructured residues of pIII and measures 18 Å (horizontal dotted line). These unstructured regions could move away to allow DNA to pass through the aperture between the helices of pIII, measured at 24 Å (vertical dotted line); the diameter of a DNA double helix is 20 Å.

(c) The pIII chains in the AlphaFold model from part (b) were overlaid with a modelled open form of pIII, where the N-terminal part of the C domain has been swung out around the β-hairpin loop (as described in Supplementary Fig. 18d). These helices would be located in the periplasm, in accordance with the MEMSAT-SVM predictions.

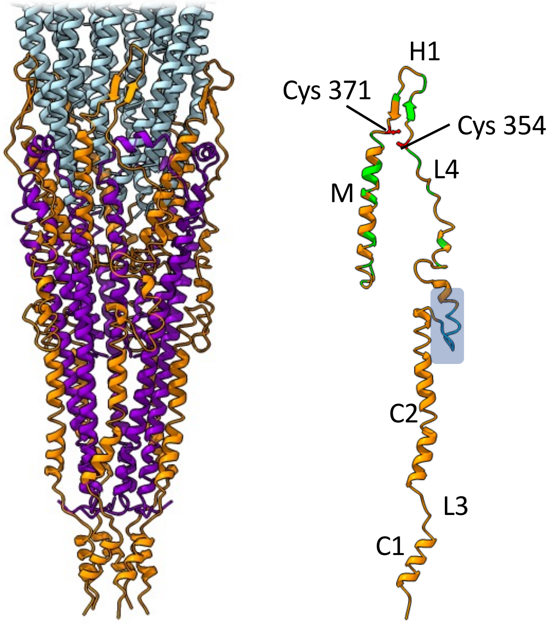

Supplementary Figure 22. Function of the C domain in assembly and egress.

Structure of the pointy tip of the page and an individual chain of pIII with domains labelled: from the N-terminus of the C domain: C-terminal domain 1 (C1), linker 3 (L3), C-terminal domain 2 (C2), linker 4 (L4), β-hairpin loop (H1) and transmembrane helix (M). The N1-N2 domains with glycine-rich linkers are not shown for clarity. Residues necessary for efficient incorporation of pIII into the wild-type coat[21] have been mapped onto our structure and shown in green. Cys 354 and Cys 371 were also shown to be essential for assembly[22] and are shown as ball-and-stick in red and labelled. The 10 amino acid long region required for termination of assembly[15] is boxed in blue.

Supplementary Tables

**Data collection and processing**			
Acceleration voltage (kV)	300		
Pixel size (Å)	1.10 (0.55 super-res)		
Defocus range (µm)	-1.3 to -2.5 (0.3 µm steps)		
Frames per movie	40		
Total dose ($e^- /Å^2$)	40		
Number of micrographs	25,065		
EMPIAR accession number	11480		
	**Pointy tip**	**Round tip**	**Central filament**
Number of particles	594,894	594,894	13,430,601
Particles in final refinement	86,242	255,372	1,201,228
Applied symmetry	C5	C5	C5<br>Helical<br>16.599 Å rise, 37.437° twist
**Model Refinement**			
PDB ID	8B3O	8B3P	8B3Q
EMDB accession number	15831	15832	15833
Resolution (Å)<br>(Masked FSC = 0.143)	2.9	2.8	2.6
**Model Composition**			
**No. of chains**			
Protein pIII	5	-	-
Protein pVI	5	-	-
Protein pVII	-	5	-
Protein pVIII	35	45	75
Protein pIX	-	5	-
Non-hydrogen atoms	20475	17635	25500
Protein residues	2450	2370	3450
Ligands	0	0	0
**RMS Deviations**			
Bond length (Å)	0.007	0.004	0.008
Bond Angle (°)	1.160	1.097	1.509
**Validation**			
Rotamer outliers (%)	2.0	1.0	0.2
**Ramachandran**			
Favoured (%)	96	100	100
Disallowed (%)	0	0	0

Supplementary Table 1. CryoEM data collection and processing details.

Virion cap composition[a]	Number of plaque forming particles (pfu/mL)[b]	Quantity of virions (ge/mL)[c]	Infectivity (pfu/ge)[d]	Relative infectivity[e]
WTpIII (+)	$5.66 \pm 0.86 \times 10^{12}$	$2.75 \pm 0.18 \times 10^{14}$	2.06×10^{-2}	1
NdC141	$2.07 \pm 0.59 \times 10^{12}$	$7.78 \pm 0.57 \times 10^{13}$	2.66×10^{-2}	1.3
NdC132	$4.98 \pm 0.34 \times 10^{12}$	$7.40 \pm 0.25 \times 10^{13}$	6.73×10^{-2}	3.27
NdC121	$2.58 \pm 0.31 \times 10^{12}$	$6.19 \pm 0.32 \times 10^{13}$	4.17×10^{-2}	2.03
NdC111	$3.57 \pm 0.66 \times 10^{9}$	$7.06 \pm 0.69 \times 10^{12}$	0.50×10^{-3}	2.46×10^{-2}
NdC93	$5.17 \pm 0.52 \times 10^{9}$	$3.72 \pm 0.27 \times 10^{12}$	1.39×10^{-3}	6.76×10^{-2}
NdC83	$6.17 \pm 1.01 \times 10^{6}$	$7.11 \pm 0.52 \times 10^{12}$	8.67×10^{-7}	4.22×10^{-5}
no pIII (-)	$4.60 \pm 1.06 \times 10^{7}$	$3.31 \pm 0.94 \times 10^{13}$	1.39×10^{-6}	6.77×10^{-5}

Supplementary Table 2. The infectivity of pIII C domain mutants.

[a]Phages were produced as described in Supplementary Methods.

[b]Titre; the number of infectious phage particles per ml.

[c]Quantity of phage particles, expressed as the number of genome equivalents (ge) per ml. A genome equivalent is a measure of particle mass and is defined as a particle containing one encapsulated genome (or the portion of a multiple length particle that contains one genome). Thus, a virion particle containing ten genomes represents ten genome equivalents, as do ten phage particles containing one genome each. The number of genome equivalents was determined from agarose gel electrophoresis of phage ssDNA, released from SDS-disassembled virions[11] as described in Supplementary Methods.

[d]Infectivity of phages, expressed as a ratio of infectious titre to quantity of phage particles.

[e]Relative infectivity expressed as the ratio of infectivity of phage particles relative to those produced in the cells expressing WT pIII (positive control).

Virion quantification and titration of the infectious particles was carried out in triplicate.

Supplementary Methods

Determination of the nanorod length from micrographs of negatively stained nanorods

Purified nanorods (10 µl of $5x10^{14}$ particles/ml) were applied onto carbon coated Cu 400 mesh grids (Electron Microscopy Sciences) for 5 min. The grid was then removed from the drop and the excess liquid was removed with filter paper before placing the grid on top of a 10 µl drop of MilliQ water for 30 sec as a washing step. Next, the grid was removed from the drop and the excess liquid was removed with filter paper. Finally, the grid was placed on top of a 10 µl drop of 2% uranyl acetate for 5 min to stain the sample, followed by the removal of the excess liquid. Images were obtained using a 120 kV Tecnai G2 Spirit BioTWIN (FEI, Czech Republic) microscope at the Manawatu Microscopy and Imaging Centre (MMIC), Palmerston North, New Zealand. Nanorod size was determined by measuring the length of 300 well-separated particles using the ImageJ[23] measurement function.

Production of C domain mutants

The host strains for phage production were obtained by transformation of the NdC141-NdC83 series[11] or the WTpIII-expressing plasmids into strain TG1 (*supE Δ(hsdM-mcrB)5* (rk^- mk^- $McrB^-$) *thi Δ(lac-proAB)* F' [*traD36 $lacI^q$ Δ(lacZ)M15 $proA^+B^+$*]). Transformants were selected for on 2xYT plates containing 100 µg/ml Amp and were colony-purified. Single colonies were used to inoculate overnight cultures for phage production. To produce phages containing WT pIII or mutants with internal C domain deletions, exponentially growing cultures were infected with phage f1d3 at a multiplicity of infection of 100 phages per cell, for 1 hour at 37 °C. Infected cells were then separated from unabsorbed phage by centrifugation (7000 x *g* for 10 minutes at 37 °C), resuspended in fresh medium containing 0.1 mM IPTG and incubated for a further period of 4 h to allow phage particle production. Following incubation, the cells were removed by centrifugation (7000 x *g* for 20 min at 30 °C) and the supernatants containing the released phages were collected for further analyses. Phages in the supernatants were concentrated by two rounds of standard PEG precipitation [PEG8000 (5 % w/v) and NaCl (0.5 M)].

Infectivity of C domain mutants

Briefly, virions were disassembled by incubation in SDS-containing buffer (1% SDS, 1× TAE buffer 5% glycerol, 0.25% BPB) at 70°C for 20 min to release the phage ssDNA and the samples were subjected to agarose gel electrophoresis. On completion of electrophoresis, phage ssDNA was stained with ethidium bromide and quantified by densitometry. Since the amount of ssDNA in a band is not linearly proportional to the intensity of the fluorescence, every gel contained a set of twofold serial dilutions of f1d3 ssDNA standard of known concentration, used for calibration. Where quantification of different sets of samples was conducted on different days (e.g. for the data in Supplementary Table 2), the same series of quantification standards (serial dilutions of purified and spectrophotometrically quantified phage ssDNA) was loaded on each gel as an internal control/standard. Quantity of phage particles was expressed as the number of genome equivalents (ge) per ml. A genome equivalent is a measure of particle mass and is defined as a particle containing one encapsulated genome (or the portion of a multiple length particle that contains one genome). Thus, a virion particle containing ten genomes represents ten

genome equivalents, as do ten phage particles containing one genome each.

Infectivity of phages was expressed as a ratio of infectious titre to quantity of phage particles. Infectious titre was determined by titration of samples on the strain TG1 as a host. For each sample the titre was determined from counts on three plates, each containing between 100 and 300 plaques. Relative infectivity was expressed as the ratio of infectivity of phage particles relative to those produced in the cells expressing WT pIII (positive control).

Testing detergent sensitivity of phages

Samples were mixed with DNA loading buffer (1× TAE, 5% glycerol, 0.25% BPB) and loaded onto 0.5% agarose gels. Different detergents were added to the loading buffer and the sample incubated at room temperature for ten minutes prior to loading. The phage particles were separated by electrophoresis at 1.3 V/cm overnight. After electrophoresis, free DNA released from phages by detergent pre-treatment was detected by staining the gel in ethidium bromide. To detect the phages, the gel was soaked in 0.2 M NaOH for 1 hour to disassemble the virions, followed by neutralisation in 0.45 M Tris pH 7.1. The exposed phage ssDNA was then visualised by staining the gel again with ethidium bromide[11].

Supplementary References

1. Sattar, S., Bennett, N. J., Wen, W. X., Guthrie, J. M., Blackwell, L. F., Conway, J. F. & Rakonjac, J. Ff-nano, short functionalized nanorods derived from Ff (f1, fd, or M13) filamentous bacteriophage. *Front. Microbiol.* **6,** 316 (2015).

2. Specthrie, L., Bullitt, E., Horiuchi, K., Model, P., Russel, M. & Makowski, L. Construction of a microphage variant of filamentous bacteriophage. *J. Mol. Biol.* **228,** 720–724 (1992).

3. Cha, T-G., Tsedev, U., Ransil, A., Embree, A., Gordon, D. B., Belcher, A. M. & Voigt, C. A. Genetic control of aerogel and nanofoam properties, applied to Ni–MnOx cathode design. *Adv. Funct. Mater.* **31,** 2010867 (2021).

4. Kestens, V., Gerganova, T., Roebben, G. & Held, A. A new certified reference material for size and shape analysis of nanorods using electron microscopy. *Anal. Bioanal. Chem.* **413**, 141-157 (2021).

5. Notredame, C., Higgins, D. G. & Heringa, J. T-Coffee: A novel method for fast and accurate multiple sequence alignment. *J. Mol. Biol.* **302,** 205–217 (2000).

6. Marvin, D. A., Welsh, L. C., Symmons, M. F., Scott, W. R. P. & Straus, S. K. Molecular structure of fd (f1, M13) filamentous bacteriophage refined with respect to X-ray fibre diffraction and solid-state NMR data supports specific models of phage assembly at the bacterial membrane. *J. Mol. Biol.* **355,** 294–309 (2006).

7. Xu, J. W., Dayan, N., Goldbourt, A. & Xiang, Y. Cryo-electron microscopy structure of the filamentous bacteriophage IKe. *Proc. Natl. Acad. Sci. USA* **116**, 5493-5498 (2019).

8. Lubkowski, J., Hennecke, F., Plückthun, A. & Wlodawer, A. The structural basis of phage display elucidated by the crystal structure of the N-terminal domains of g3p. *Nat. Struct. Biol.* **5,** 140–147 (1998).

9. Holliger, P., Riechmann, L. & Williams, R. L. Crystal structure of the two N-terminal domains of g3p from filamentous phage fd at 1.9 A: evidence for conformational lability. *J. Mol. Biol.* **288,** 649–657 (1999).

10. Tunyasuvunakool, K. *et al.* Highly accurate protein structure prediction for the human proteome. *Nature* **596**, 590-596 (2021).

11. Bennett, N. J., Gagic, D., Sutherland-Smith, A. J. & Rakonjac, J. Characterization of a dual-function domain that mediates membrane insertion and excision of Ff filamentous bacteriophage. *J. Mol. Biol.* **411,** 972–985 (2011).

12. Rakonjac, J., Jovanovic, G. & Model, P. Filamentous phage infection-mediated gene expression: Construction and propagation of the gIII deletion mutant helper phage R408d3. *Gene* **198**, 99-103 (1997).

13. Bennett, N. J. & Rakonjac, J. Unlocking of the filamentous bacteriophage virion during infection is mediated by the C domain of pIII. *J. Mol. Biol.* **356,** 266–273 (2006).

14. Crissman, J. W. & Smith, G. P. Gene-III protein of filamentous phages: evidence for a carboxyl-terminal domain with a role in morphogenesis. *Virology* **132**, 445-455 (1984).

15. Rakonjac, J., Feng, J. N. & Model, P. Filamentous phage are released from the bacterial membrane by a two-step mechanism involving a short C-terminal fragment of pIII. *J. Mol. Biol.* **289**, 1253-1265 (1999).

16. Nugent, T. & Jones, D. T. Transmembrane protein topology prediction using support vector machines. *BMC*

Bioinformatics **10,** 159 (2009).

17. Buchan, D. W. A. & Jones, D. T. The PSIPRED Protein Analysis Workbench: 20 years on. *Nucleic Acids Res.* **47,** W402–W407 (2019).

18. Lubkowski, J., Hennecke, F., Plückthun, A. & Wlodawer, A. Filamentous phage infection: crystal structure of g3p in complex with its coreceptor, the C-terminal domain of TolA. *Structure* **7,** 711–722 (1999).

19. Macé, K., Vadakkepat, A. K., Redzej, A., Lukoyanova, N., Oomen, C., Braun, N., Ukleja, M., Lu, F., Costa, T. R. D., Orlova, E. V., Baker, D., Cong, Q. & Waksman, G. Cryo-EM structure of a type IV secretion system. *Nature* **607**, 191-196 (2022).

20. Costa, T. A. R., Ilangovan, A., Ukleja, M., Smith, T. K., Egelman, E. H. & Waksman, G. Structure of the bacterial sex F pilus reveals an assembly of a stoichiometric protein-phospholipid complex. *Cell* **166,** 1436-1438 (2016).

21. Weiss, G. A., Roth, T. A., Baldi, P. F. & Sidhu, S. S. Comprehensive mutagenesis of the C-terminal domain of the M13 gene-3 minor coat protein: The requirements for assembly into the bacteriophage particle. *J. Mol. Biol.* **332,** 777–782 (2003).

22. Kremser, A. & Rasched, I. The adsorption protein of filamentous phage fd: assignment of its disulfide bridges and identification of the domain incorporated in the coat. *Biochemistry* **33,** 13954–13958 (1994).

23. Schneider, C. A., Rasband, W. S. & Eliceiri, K. W. NIH Image to ImageJ: 25 years of image analysis. *Nat. Methods* **9,** 671–675 (2012).
