# Supplementary material for: Cryo-electron microscopy of the f1 filamentous phage reveals insights into viral infection and assembly
Source: Nat Commun. 2023 May 11;14:2724. doi: 10.1038/s41467-023-37915-w (PMC10175506; doi:10.1038/s41467-023-37915-w)
Supplement: Supplementary file 3 — Description of Additional Supplementary Files [file 41467_2023_37915_MOESM3_ESM.pdf]

<u>Description of additional Supplementary information files</u>

Supplementary movie 1

CryoEM map and atomic model of the pointy tip (proteins pIII/pVI/pVIII) from the f1 filamentous bacteriophage.

Supplementary movie 2

CryoEM map and atomic model of the round tip (proteins pVII/pIX/pVIII) from the f1 filamentous bacteriophage.

Supplementary movie 3

CryoEM map and atomic model of the central filamentous region (protein pVIII) from the f1 filamentous bacteriophage.

Supplementary movie 4

Model of a short version of an assembled phage created by aligning the pointy and round tip models. The model contains 65 copies of pVIII and is ~38 nm long. WT phage contains ~2,700 copies of pVIII and is ~1µm long. WT phage would therefore be ~26x longer than the short model shown.

Supplementary movie 5

Morph of the pIII N1-N2 domains from the closed (unbound) to the open (post F-pilus binding) state. The closed state was obtained from PDB 1G3P, and the open state was made by manually opening the flexible hinge region, guided by the position of N1 bound to TolA in PDB 1TOL.

Supplementary movie 6

Proposed model for pointy tip opening and insertion into the cytoplasmic membrane. The N1-N2 domains of pIII are not shown for clarity. For pIII, the morph was generated from the phage assembled state of the protein, transitioning to the model where the C-domain has been manually swung out around the β-hairpin. For pVI, the morph was generated from the phage assembled state of the protein, transitioning to the pVI Alphafold model, followed by the Alphafold model of the pVI pentameric complex.
