# Supplementary material for: Cryo-electron microscopy of the f1 filamentous phage reveals insights into viral infection and assembly
Source: Nat Commun. 2023 May 11;14:2724. doi: 10.1038/s41467-023-37915-w (PMC10175506; doi:10.1038/s41467-023-37915-w)
Supplement: Supplementary file 10 — Reporting Summary [file 41467_2023_37915_MOESM10_ESM.pdf]

nature portfolio

Corresponding author(s): Vicki Gold
Jasna Rakonjac

Last updated by author(s): 13.03.23

Reporting Summary

Nature Portfolio wishes to improve the reproducibility of the work that we publish. This form provides structure for consistency and transparency in reporting. For further information on Nature Portfolio policies, see our Editorial Policies and the Editorial Policy Checklist.

Statistics

For all statistical analyses, confirm that the following items are present in the figure legend, table legend, main text, or Methods section.

n/a	Confirmed	
☐	☒	The exact sample size (*n*) for each experimental group/condition, given as a discrete number and unit of measurement
☐	☒	A statement on whether measurements were taken from distinct samples or whether the same sample was measured repeatedly
☒	☐	The statistical test(s) used AND whether they are one- or two-sided<br>*Only common tests should be described solely by name; describe more complex techniques in the Methods section.*
☒	☐	A description of all covariates tested
☒	☐	A description of any assumptions or corrections, such as tests of normality and adjustment for multiple comparisons
☒	☐	A full description of the statistical parameters including central tendency (e.g. means) or other basic estimates (e.g. regression coefficient) AND variation (e.g. standard deviation) or associated estimates of uncertainty (e.g. confidence intervals)
☒	☐	For null hypothesis testing, the test statistic (e.g. *F*, *t*, *r*) with confidence intervals, effect sizes, degrees of freedom and *P* value noted<br>*Give P values as exact values whenever suitable.*
☒	☐	For Bayesian analysis, information on the choice of priors and Markov chain Monte Carlo settings
☒	☐	For hierarchical and complex designs, identification of the appropriate level for tests and full reporting of outcomes
☒	☐	Estimates of effect sizes (e.g. Cohen's *d*, Pearson's *r*), indicating how they were calculated

Our web collection on statistics for biologists contains articles on many of the points above.

Software and code

Policy information about availability of computer code

Data collection	EPU 2.8.1
Data analysis	Image J 1.5<br>Warp 1.0.9<br>cryoSPARC 3.2.0<br>ChimeraX 1.3<br>Coot 0.9.3<br>Refmac 5 (CCPEM suite)<br>CCPEM 1.4.2<br>Molprobity 4.2<br>DeepEMhancer (Sep 2020)<br>AlphaFold2<br>T-Coffee<br>MEMSAT-SVM 1.3

For manuscripts utilizing custom algorithms or software that are central to the research but not yet described in published literature, software must be made available to editors and reviewers. We strongly encourage code deposition in a community repository (e.g. GitHub). See the Nature Portfolio guidelines for submitting code & software for further information.

Data

Policy information about availability of data

All manuscripts must include a data availability statement. This statement should provide the following information, where applicable:

- Accession codes, unique identifiers, or web links for publicly available datasets
- A description of any restrictions on data availability
- For clinical datasets or third party data, please ensure that the statement adheres to our policy

The 3D cryoEM density maps generated in this study have been deposited in the Electron Microscopy Data Bank (EMDB) under accession codes EMD-15831, EMD-15832 and EMD-15833 for pointy, round and central filamentous maps respectively. The atomic coordinates have been deposited in the Protein Data Bank (PDB) under accession numbers 8B3O, 8B3P and 8B3Q. The source image data used in this study have been deposited to the Electron Microscopy Public Image Archive (EMPIAR) under accession number EMPIAR-11480. The previously determined structures of the fd pVIII (2C0W), N1 domain of pIII bound to TolA (1TOL), N1-N2 domains of pIII (1G3P), the F-pilus (5LER), the stalk of the type IV secretion system (7O3V) used in this study are available in the PDB under accession codes [http://doi.org/10.2210/pdb2c0w/pdb] (fd), [http://doi.org/10.2210/pdb1TOL/pdb] (N1-TolA), [http://doi.org/10.2210/pdb1G3P/pdb] (N1-N2), [http://doi.org/10.2210/pdb5LER/pdb] (F-pilus), and [http://doi.org/10.2210/pdb7O3V/pdb] (stalk). Uncropped versions of the nanorod purification gels (Supplementary Fig. 2), native agarose gel electrophoresis (Supplementary Fig. 17) and the data corresponding to the infectivity of pIII C domain mutants (Supplementary Table 2) are provided in the Source Data file.

Human research participants

Policy information about studies involving human research participants and Sex and Gender in Research.

Reporting on sex and gender	N/A
Population characteristics	N/A
Recruitment	N/A
Ethics oversight	N/A

Note that full information on the approval of the study protocol must also be provided in the manuscript.

Field-specific reporting

Please select the one below that is the best fit for your research. If you are not sure, read the appropriate sections before making your selection.

[x] Life sciences [ ] Behavioural & social sciences [ ] Ecological, evolutionary & environmental sciences

For a reference copy of the document with all sections, see nature.com/documents/nr-reporting-summary-flat.pdf

Life sciences study design

All studies must disclose on these points even when the disclosure is negative.

Sample size	A single nanorod purification (Supplementary Fig. 2) was used for structural analysis. For cryoEM structure determination, sample size was determined by the availability of particles on the grid.<br><br>The quantification in Supplementary Fig. 2d and e contained a sample size of 300. The sample size was based on statistical considerations adopted for certification of nanomaterials (presented by a report of the European Commission, Joint Research Centre Directorate F – Health, Consumers and Reference Materials, Geel, Belgium; https://publications.jrc.ec.europa.eu/repository/handle/JRC117117) used to assess dispersity of particles in nanorod standards by TEM.<br><br>Each of the f1d3 filamentous phage preparations analysed in Supplementary Fig. 17 and Supplementary Table 2 were derived from three technical replicates with similar results to ensure reproducibility.
Data exclusions	Classification in cryoEM image processing was used to remove poorly aligned particles.

Replication	A single nanorod purification (Supplementary Fig. 2) was used for structural analysis. Similar experiments were conducted at least 3 times with similar results. The structural experiments were not replicated as these are not high throughput and it is not standard practice in the field to repeat these experiments multiple times.<br><br>Each of the f1d3 filamentous phage preparations analysed in Supplementary Fig. 17 and Supplementary Table 2 were derived from three technical replicates with similar results to ensure reproducibility. The reproducibility of the pattern of phage bands in native agarose gel electrophoresis and sensitivity to sarkosyl (Supplementary Fig. 17) is independently confirmed by comparison to the published pattern of the f1d3 virion sizes and sarkosyl sensitivity when virions were produced in the presence of the equivalent series C-terminal pIII fragments (but lacking the N1N2 domains (Rakonjac et al, F Mol Biol (1999) https://pubmed.ncbi.nlm.nih.gov/10373366/).
Randomization	Samples did not need to be allocated into different experimental groups as this was not relevant to our study.
Blinding	Blinding is not possible for this type of study.

Reporting for specific materials, systems and methods

We require information from authors about some types of materials, experimental systems and methods used in many studies. Here, indicate whether each material, system or method listed is relevant to your study. If you are not sure if a list item applies to your research, read the appropriate section before selecting a response.

Materials & experimental systems

n/a	Involved in the study
☒	☐ Antibodies
☒	☐ Eukaryotic cell lines
☒	☐ Palaeontology and archaeology
☒	☐ Animals and other organisms
☒	☐ Clinical data
☒	☐ Dual use research of concern

Methods

n/a	Involved in the study
☒	☐ ChIP-seq
☒	☐ Flow cytometry
☒	☐ MRI-based neuroimaging
